# Prevalence of Drug Resistance Associated Substitutions in Persons With Chronic Hepatitis C Infection and Virological Failure Following Initial or Re-treatment With Pan-genotypic Direct-Acting Antivirals: A Systematic Review and Meta-analysis

**DOI:** 10.1093/cid/ciae431

**Published:** 2024-10-03

**Authors:** Seth Inzaule, Philippa Easterbrook, Ashley Latona, Nathan P Ford, William Irving, Philippa C Matthews, Marco Vitoria, Chris Duncombe, Amalia Giron, Suzanne McCluskey, Olufunmilayo Lesi, Serge Tchamgoue, Rachel Halford, Danjuma Adda, Emma Thomson, Geoff Dusheiko, Michael R Jordan

**Affiliations:** Amsterdam Institute for Global Health and Development, and Department of Global Health, Amsterdam UMC, University of Amsterdam, Amsterdam, The Netherlands; HIV, Hepatitis and Sexually Transmitted Infection Department, World Health Organization, Geneva, Switzerland; Division of Geographic Medicine and Infectious Diseases,Tufts University School of Medicine, Boston, Massachusetts, USA; HIV, Hepatitis and Sexually Transmitted Infection Department, World Health Organization, Geneva, Switzerland; School of Life Sciences, Division of Microbiology and Infectious Diseases, The University of Nottingham, Nottingham, United Kingdom; The Francis Crick Institute, London, United Kingdom; HIV, Hepatitis and Sexually Transmitted Infection Department, World Health Organization, Geneva, Switzerland; International Association of Providers of AIDS Care, Washington, DC, USA; Independent Consultant, Guatemala city, Guatemala; Division of Infectious Diseases, Havard Medical School, Massachusetts General Hospital, Boston, Massachusetts, USA; HIV, Hepatitis and Sexually Transmitted Infection Department, World Health Organization, Geneva, Switzerland; Faculty of Medicine and Pharmaceutical Sciences, University of Douala, Douala, Cameroon; World Hepatitis Alliance, Geneva, Switzerland; World Hepatitis Alliance, Geneva, Switzerland; Medical Research Council-University of Glasgow Centre for Virus Research, Glasgow, United Kingdom; Institute for Global Health, University College London, London, United Kingdom; Division of Geographic Medicine and Infectious Diseases,Tufts University School of Medicine, Boston, Massachusetts, USA

**Keywords:** pan-genotypic direct acting agents, treatment failure, resistance-associated substitutions, chronic hepatitis C virus, initial treatment and retreatment

## Abstract

**Background:**

The advent of short-course, curative treatment with direct-acting antivirals (DAA) has given promise for the global elimination of hepatitis C virus (HCV) infections by 2030. Virological failure occurs in 2%–12% of persons receiving curative DAA treatment and may be presaged by pre-existing polymorphisms or result from selection of drug resistant variants during therapy.

**Methods:**

We conducted a systematic review to assess the prevalence of HCV resistance associated substitutions (RAS) among individuals with chronic hepatitis C infection who had virological failure following initial or re-treatment with pan-genotypic DAA regimens. We included 34 and 22 studies assessing RAS in people with virological failure published between January 2014 and July 2023. Pooled RAS prevalence was estimated using random-effects meta-analysis.

**Results:**

The pooled prevalence of RAS in people with virological failure following initial DAA treatment was 78.0% (95% confidence interval [CI]: 62.0–92.0) for sofosbuvir/velpatasvir, 81.0% (95% CI: 67.0–93.0) for sofosbuvir/daclatasvir, and 79.0% (95% CI: 70.0–87.0) for glecaprevir/pibrentasvir, with a high prevalence of resistance to the NS5A inhibitors. Among those with virological failure following re-treatment regimens, RAS were present in 93.0% (95% CI: 83.0–99.0) for sofosbuvir/velpatasvir/voxilepravir and in 100% (95% CI: 92.0–100) for glecaprevir/pibrentasvir, with resistance driven by RAS to NS5A inhibitors.

**Discussion:**

At least 1 RAS is present in a high proportion of the few individuals with virological failure following initial or re-treatment with pan-genotypic DAA regimens. There is a need for ongoing surveillance for DAA-associated resistance, to assess risk factors for their development and clinical impact to inform best practice strategies for re-treatment.

## Teaser

Direct-acting antiviral (DAA)-associated resistance is common among individuals with chronic hepatitis C virus failing initial or re-treatment pan-genotypic DAAs. There is need for surveillance of DAA-associated resistance, to assess risk factors for their selection, and clinical impact to inform re-treatment regimen selection.

The introduction of oral direct-acting antivirals (DAA) in 2014, which provide potent and well-tolerated short-course curative treatment, has revolutionized hepatitis C virus (HCV) management and given promise for the global elimination of HCV infection [1].

World Health Organization (WHO) recommends dual-DAA combination therapy targeting at least 2 distinct non-structural proteins. The recommended pan-genotypic regimens for the initial treatment consist of the NS5B nucleoside polymerase inhibitor (NPI) sofosbuvir (SOF) in combination with either the NS5A replication complex inhibitors (NS5A) daclatasvir (DAC) or velpatasvir (VEL) or a dual combination of the NS5A inhibitor, glecaprevir (GLE) with the NS3/4A polymerase inhibitor (NS3/4A) PI pibrentasvir (PIB) [2, 3].

The pan-genotypic DAAs recommended by WHO are highly effective with cure rates (defined as sustained virological response at 12 weeks after completion of treatment) of generally >95%; however, around 2% to 12% experience virological failure [4–12]. Factors associated with treatment failure include prior treatment experience with interferon regimens, poor adherence to treatment, presence of cirrhosis, and pre-existing resistance-associated substitutions (RAS), or polymorphisms linked to certain subtypes which confer lower sensitivity to the existing DAAs [13, 14]. Virological failure is often associated with selection of RAS related to the drugs used in the therapeutic regimen [15–17]. Although there are concerns that RAS may limit retreatment options, some studies indicate that increasing the duration of treatment with the same regimen or alternative regimens may achieve cure [18–20]. Currently, only 1 pan-genotypic regimen (sofosbuvir [SOF]/velpatasvir [VEL]/voxilaprevir [VOX]) targeting 3 drug target sites is recommended by several professional societies for retreatment after failure of initial DAA regimens [2, 3, 21, 22]. The pan-genotypic combination GLE/PIB administered alone or in combination with SOF is also proposed for use in those with virological failure following use of SOF-containing regimens and or either a PI or an NS5A inhibitor (but not both) [2, 3].

Over the last 10 years, there have been considerable efforts to scale up HCV diagnosis and expand access to treatment in low- and middle-income countries (LMICs). As of 2022, WHO estimates that 36% of those with chronic hepatitis C infection have been diagnosed, and 20% treated. However, progress has been uneven, and diagnosis and treatment coverage is lower in low- and middle-income countries, especially in Sub-Saharan Africa (SSA) (<1%) and Southeast Asia (5%) [1]. The availability of generic DAAs, particularly the combinations of SOF/DAC and SOF/VEL, has helped improve access to treatment due to their lower cost [23]. Although treatment failure rates are low ranging from 2% to 12% [22], there are concerns that in settings and populations with a high burden of infection and/or where there are also ongoing high rates of transmission [24], such treatment failures may be consequential, particularly in the presence of clinically significant resistance [22]. Overall, the potential for selection of RAS during virological failure and possible subsequent onward transmission [15–17] in the context of limited options for retreatment regimens in LMICs needs attention. Information on prevalence of DAA-associated drug resistance mutations in those with virological failure following initial DAA treatment or re-treatment and their clinical significance remains scarce in resource-limited settings and is important to inform re-treatment and appropriate prevention and monitoring strategies for LMICs.

We conducted a systematic review to determine the prevalence of RAS among persons with chronic hepatitis C infection with virological failure after initial or re-treatment with pan-genotypic DAA regimens.

## METHODS

### Search Strategy and Selection Criteria

We conducted a systematic review by searching the available English language literature in PubMed in accordance with PRISM guidelines following a predefined study protocol [25]. The search terms and procedures are described in Supplementary Appendix pp1.

Two authors (S. C. and A. L.) independently screened titles and abstracts from the search and subsequently screened the full texts of potentially eligible articles. We included prospective cross-sectional studies, prospective observational studies, and randomized trials but excluded case studies and studies that did not clearly report a denominator. We assessed the risk of bias for each record using a modified Johanna Briggs institute critical appraisal tool for systematic reviews of prevalence studies [26] (Supplementary Appendix). Our primary outcome was the prevalence of RAS following either treatment failure (ie, failure to achieve SVR after treatment duration) or breakthrough infection (ie, having detectable HCV RNA on treatment after initially developing undetectable HCV RNA levels during treatment). RAS was defined as any clinically relevant mutation based on EASL 2020 mutation list [22] or by the geno-2-pheno database [27].

To estimate the prevalence of RAS after initial treatment failure to WHO-recommended pan-genotypic regimens we included data from people experiencing virological failure defined as having viral breakthrough or viral relapse after treatment with either SOF/VEL, SOF/DAC, or GLE/PIB with or without co-administration of ribavirin. We excluded studies or subjects who had a history of prior DAA treatment but included those who had been pre-treated with pegylated interferon and/or ribavirin before starting DAA treatment. We also excluded studies that did not report RAS at time of failure.

For assessment of the prevalence of RAS in people experiencing failure after retreatment, we included studies from people in whom prior DAA treatment had failed and who were being retreated with SOF/VEL/VOX or GLE/PIB. We excluded studies using SOF/VEL/VOX or GLE/PIB as initial treatment and those that did not report RAS information. We extracted RAS prevalence information, as well as study characteristics (eg, study design and region) and HCV genotypes in Microsoft Excel. Reported RAS were re-analyzed using the EASL 2020 drug resistance mutations list [22]. In cases where analyses had been done based on the geno-2-pheno resistance algorithm [27], no additional re-analysis was performed. In studies that also reported the presence of RAS present at low abundance, we only included RAS in our analysis if they were reported at a threshold of ≥15% as recommended by other treatment guidelines [21, 28] because the clinical relevance of low abundant RAS remains uncertain [29].

#### Data Analyses

The proportions and corresponding 95% confidence intervals (CIs) of RAS were first transformed using Freeman–Tukey Double Arcsine transformation to stabilize on the variances due to sparse data [30]. The pooled prevalence was calculated using random effects meta-analysis because high between-study variability was expected. We evaluated between-study heterogeneity using T^2^ statistic. We also estimated pooled RAS prevalence by HCV genotype (GT) in cases where we had at least 2 studies.

## Supplementary Material

ciae431_Supplementary_Data

## Figures and Tables

**Figure 1.**
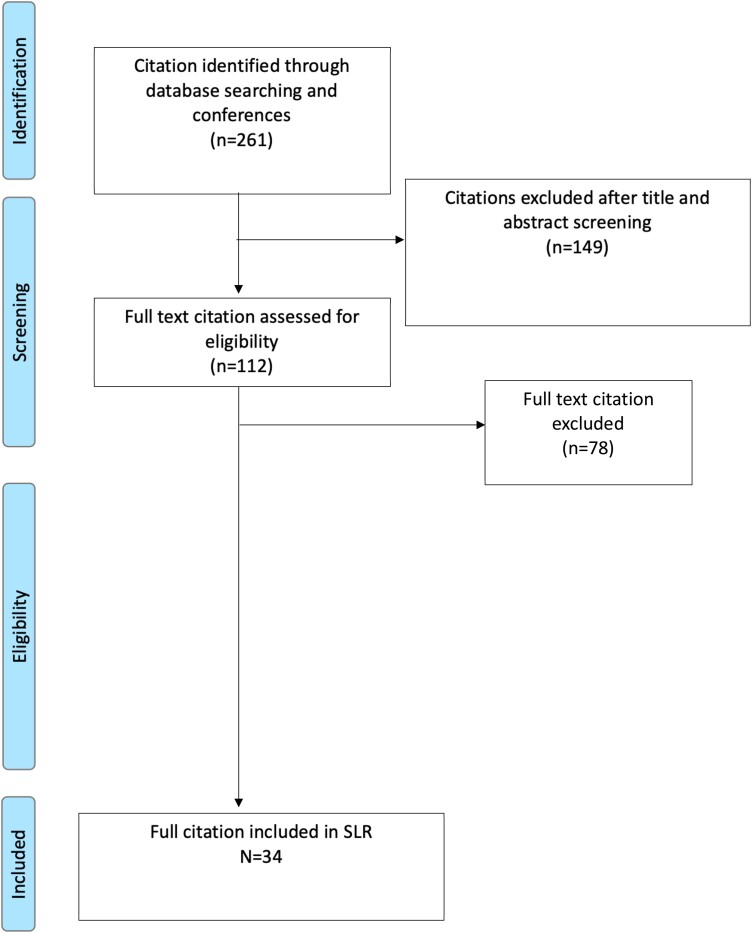
Study selection among patients with viral non-suppression after WHO recommended first-line direct acting antiviral treatment of hepatitis C. Abbreviation: WHO, World Health Organization.

**Figure 2.**
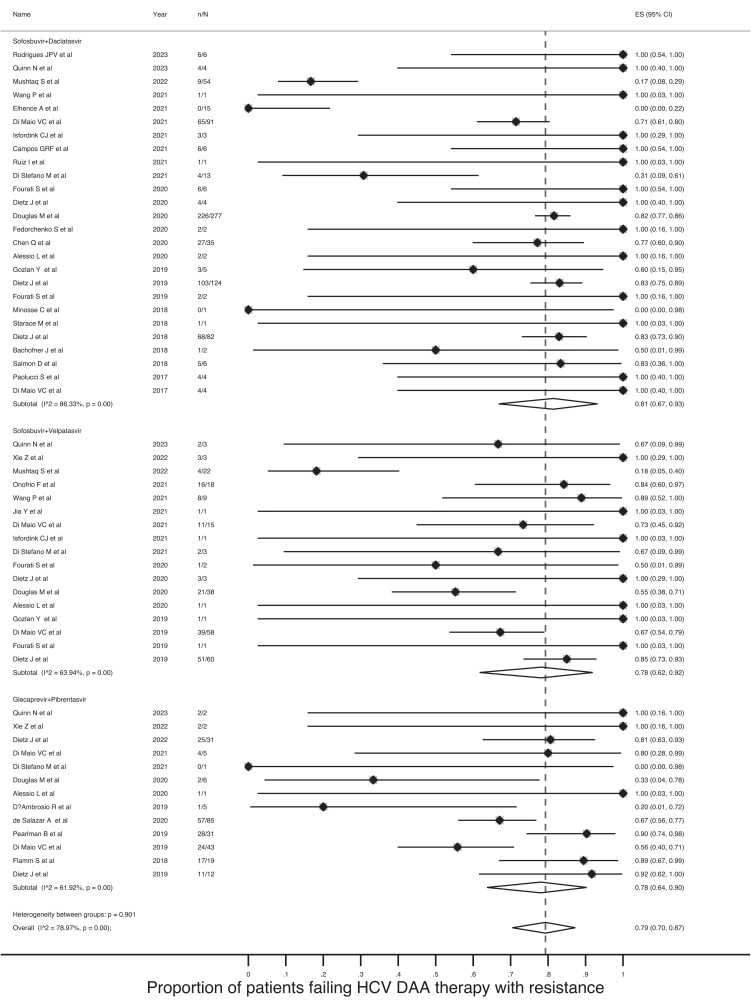
Prevalence of post-treatment RAS among patients treated with SOF/DAC, SOF/VEL, or GLE/PIB as initial regimen. Abbreviations: CI, confidence interval; DAC, daclatasvir; ES, effect size (odds ratio); GLE, glecaprevir; I^2^, heterogeneity quantified using the I^2^ statistic; PIB, pibrentasvir; RAS, resistance associated substitution; SOF, sofosbuvir; VEL, velpatasvir.

**Table 1.** Prevalence of HCV Resistance Associated Substitutions Among Patients in Whom WHO-recommended First-line DAA Treatment is Failing

DAA Regimen	No. of Studies	Drug Target Region (%, 95% CI)			
		All	NS5A	NS5B	NS53/4A
SOF/VEL	17	78.0 (62.0–92.0)	72.0 (55.0–88.0)	0.0 (0.0–8.0)^a^	…
SOF/DAC	26	81.0 (67.0–93.0)	81.0 (66.0–93.0)	0.0 (0.0–10.0)^b^	…
GLE/PIB	13	78.0 (64.0–90.0)	72.0 (57.0–85.0)	…	22.0 (8.0–39.0)

Abbreviations: CI, confidence interval; DAC, daclatasvir; GLE, glecaprevir; PIB, pibrentasvir; SOF, sofosbuvir; VEL, velpatasvir.

^a^Reported by only 13 of 17 studies.

^b^Reported by only 24 of 26 studies.

**Table 2.** Prevalence of Resistance Associated Substitutions Among Patients in Whom WHO-recommended First-line DAA Treatment Is Failing Across HCV Genotypes

	GT1		GT2		GT3		GT4		GT5		GT6	
	N^a^	% (95% CI)	N^a^	% (95% CI)	N^a^	% (95% CI)	N^a^	% (95% CI)	N^a^	% (95 % CI)	N^a^	% (95% CI)
SOF/VEL	5	74.0 (52.0–92.0)	1	40 (12.2–73.8)^b^	10	72.0 (44.0–94.0)	2	88.0 (37.0–100)	0	…	2	100 (21.0–100)
SOF/DAC	10	90.0 (68.0–100)	2	100 (21.0–100)	18	80.0 (62.0–95.0)	3	91.0 (26.0–100)	0	…	1	0 (0–97.5)^b^
GLE/PIB	4	96.0 (82.0–100)	3	23.0 (9.0–39.0)	10	80.0 (66.0–92.0)	1	0 (0–97.5)^b^	0	…	0	

Abbreviations: CI, confidence interval; DAA, direct-acting antiviral; DAC, daclatasvir; GLE, glecaprevir; HCV, hepatitis C virus; PIB, pibrentasvir; SOF, sofosbuvir; VEL, velpatasvir; WHO, World Health Organization.

^a^Number of studies.

^b^Included only 1 study and thus no meta-analyses was performed.

**Figure 3.**
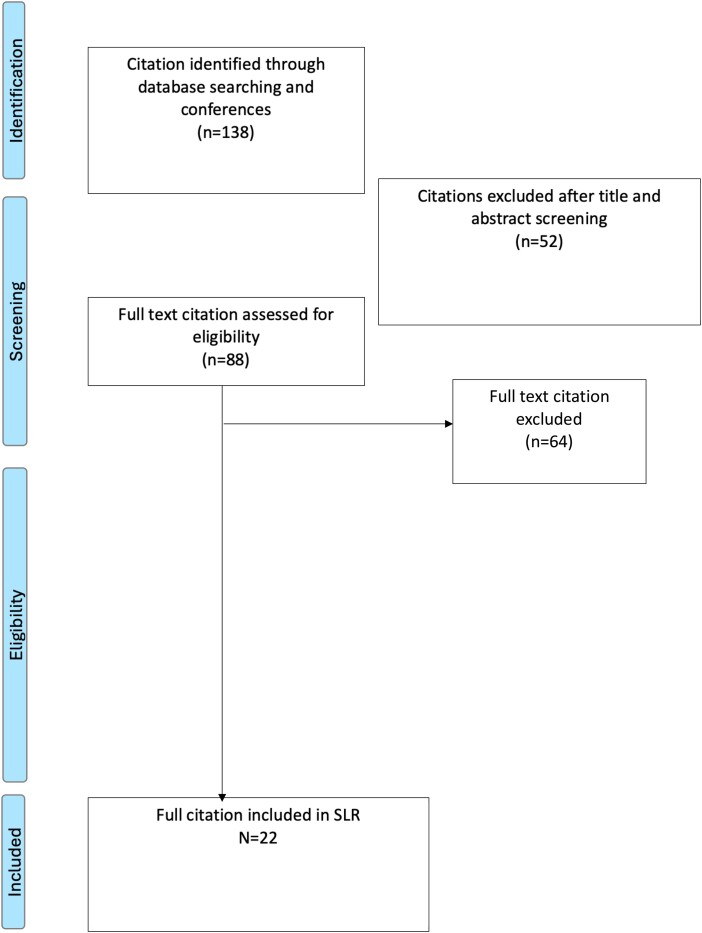
Study selection among patients with viral non-suppression after retreatment with WHO recommended second-line direct acting antiviral treatment of hepatitis C. Abbreviation: WHO, World Health Organization.

**Figure 4.**
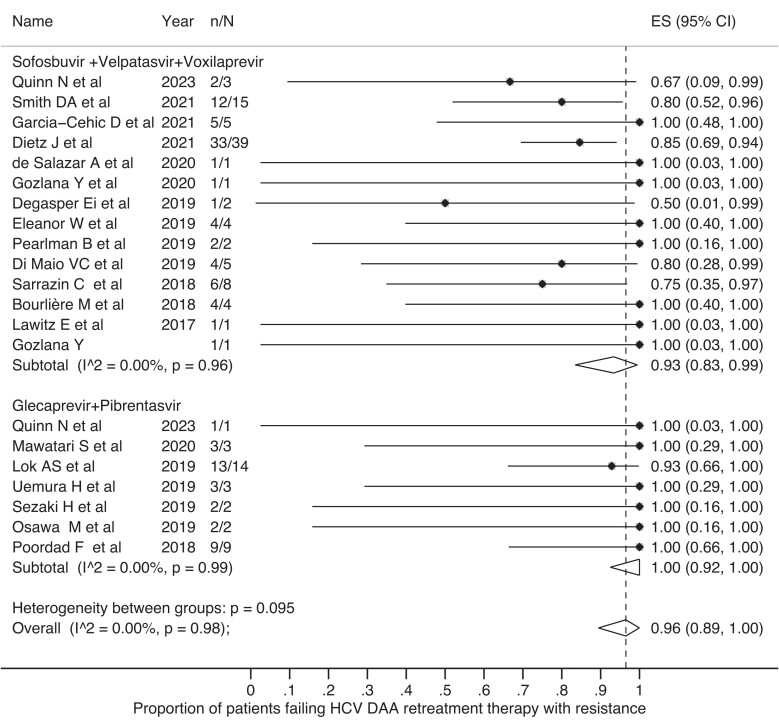
Prevalence of post-treatment RAS among patients re-treated with SOF/VEL/VOX or GLE/PIB. CI, confidence interval; DAC, daclatasvir; ES, effect size (odds ratio); GLE, glecaprevir; I^2^, heterogeneity quantified using the I^2^ statistic; PIB, pibrentasvir; SOF, sofosbuvir; VEL, velpatasvir; VOX, voxilaprevir.

**Table 3.** Prevalence of HCV Resistance Associated Substitutions Among Patients in Whom WHO-recommended Second-line DAA Treatment Is Failing

DAA Regimen	No. of Studies	Drug Target Region (%, 95% CI)			
		All	NS5A	NS5B	NS53/4A
SOF/VEL/VOX	13	93.0 (83.0–99.0)	84.0 (73.0–93.0)	6.0 (0.0–27.0)^a^	50.0 (27.0–74.0)^a^
GLE/PIB	7	100.0 (92.0–100)	97.0 (82.0–100)	…	56.0 (34.0–76.0)

Abbreviations: CI, confidence interval; DAA, direct-acting antiviral; GLE, glecaprevir; HCV, hepatitis C virus; SOF, sofosbuvir; PIB, pibrentasvir; VEL, velpatasvir; VOX, voxilaprevir; WHO, World Health Organization.

^a^Reported only by 11 of 13 studies.

**Table 4.** Prevalence of Resistance Associated Substitutions Among Patients in Whom WHO-recommended Second-line DAA Treatment Is Failing Across HCV Genotypes

	GT1		GT2		GT3		GT4		GT5		GT6	
	N^a^	% (95%CI)	N^a^	% (95%CI)	N^a^	% (95%CI)	N^a^	% (95%CI)	N^a^	% (95%CI)	N^a^	% (95%CI)
SOF/VEL/VOX	11	90.0 (74.0–100.0)	0	…	8	95.0 (79.0–100)	4	82.0 (17.0–100)	0	…	0	…
GLE/PIB	7	100.0 (92.0–100)	0	…	0	…	0	…	0	…	0	…

Abbreviations: CI, confidence interval; DAA, direct-acting antiviral; GLE, glecaprevir; HCV, hepatitis C virus; PIB, pibrentasvir; SOF, sofosbuvir; VEL, velpatasvir; VOX, voxilaprevir; WHO, World Health Organization.

^a^Number of studies.

## RESULTS

### Prevalence of Post-treatment RAS Among Patients Treated With SOF/DAC, SOF/VEL or GLE/PIB

Of 261 potentially eligible publications assessing the prevalence of RAS in those with virological failure following initial DAA treatment, 34 studies comprising 1234 patients were included (Figure 1). These included 26 studies assessing RAS among those where the initial regimen was SOF/DAC, 17 studies where initial regimen was SOF/VEL, and 13 where initial regimen was GLE/PIB. The characteristics of the included studies are shown in Supplementary Tables 1, 2, and 3 and quality of evidence rating in Supplementary Tables 6–8. Most studies reported data on GT1 and GT3. Data were rated as limited for GT2 and severely limited for GT4, 5, 6 and 7 (Supplementary Tables 1–3, 12). Most studies were from the WHO European Region (18/34); and importantly there were no studies from the WHO African region (Supplementary Tables 1–3, 11).

The overall pooled prevalence of RAS among people not cured by the initial DAA treatment was 79.0% (95% CI: 70.0%–87.0%) overall; 81% (95% CI: 67%–93%) for those treated with SOF/DAC, 78.0% (95% CI: 62.0%–92.0%) for SOF/VEL, and 78.0% (95% CI: 64.0%–90.0%) for GLE/PIB (Figure 2, Table 1). Among those receiving SOF-based regimens, RAS prevalence was driven by high-level resistance to NS5A inhibitor drugs: 81.0% (95% CI: 66.0–93.0) for DAC-containing regimens and 72.0% (95% CI: 55.0–88.0) for the SOF/VEL combination (Table 1). Resistance to SOF was rare, estimated at 0% (95% CI: .0–8.0) for those receiving SOF in combination with DAC, and 0% (95% CI: .0–10) for those receiving a VEL-containing regimen (Table 1). For participants with virological failure following GLE/PIB, drug resistance was similarly driven by high-level resistance to the NS5A inhibitor PIB (72.0%, 95% CI: 57.0–85.0), whereas resistance to the NS3/4A PI GLE was 22% (95% CI: 8.0–39.0) (Table 1). Data on prevalence of drug resistance by genotype are shown in Table 2, whereas frequency of mutations by genotype is shown in Supplementary tables 15–17.

Of 138 potentially eligible studies assessing RAS in people with virological failure after retreatment, 22 eligible studies comprising a total of 124 patients were included in the analyses (Figure 3). Of these, 13 studies were in patients receiving SOF/VEL/VOX, and 7 in patients receiving GLE/PIB. The characteristics of the included studies are shown in Supplementary Tables 4–5 and quality of evidence in Supplementary Tables 9–10. Most of the studies were conducted in the WHO European region, the Region of the Americas, and the Western Pacific region. There were no retreatment studies from the WHO African region (Supplementary Table 13). All studies assessing GLE/PIB retreatment were from patients infected with GT1, whereas studies assessing retreatment outcomes among patients receiving SOF/VEL/VOX were mostly from patients infected with either GT1 (11) or GT3 (8), with only few studies reporting on those infected with GT4 (4) and none from those infected with GT2, 5, 6, or 7 (Supplementary Table 14).

### Prevalence of Post-treatment RAS Among Patients Re-treated With SOF/VEL/VOX or GLE/PIB

The overall pooled prevalence of RAS among people not cured by the retreatment DAA regimen was 96.0% (95% CI: 89%–100%), being 93.0% (95% CI: 83.0%–99.0%) after SOF/VEL/VOX retreatment, and 100% (95% CI: 89.0%–100%) after GLE/PIB retreatment (Figure 4, Table 3). Among those with SOF/VEL/VOX failure, the high levels of RAS were mainly to the NS5A inhibitor VEL, 84.0% (95% CI: 73.0–93.0) (Table 3). The prevalence of resistance to the NS3/4A PI, VOX was 50.0% (95% CI: 27.0–74.0), and to the NS5B inhibitor, SOF was 6.0% (95% CI: .0–27.0) (Table 3). For patients with GLE/PIB virological failure, resistance was similarly driven by high levels of RAS to the NS5A inhibitor PIB; 97.0% (95% CI: 82.0–100), whereas RAS to the NS3/4A PI, GLE was 56.0% (95% CI: 34.0–76.0) (Table 3). Data on the prevalence of RAS by HCV genotype is shown in Table 4, and the frequency of mutations by genotype is shown in Supplementary Tables 18–19.

## DISCUSSION

We report a high prevalence of RAS in persons with chronic hepatitis C infection with virological failure following either initial treatment or re-treatment with WHO-recommended pan-genotypic DAA regimens. Overall, drug resistance was driven by RAS to NS5A inhibitors and was higher in those with virological failure following re-treatment, with only a few patients exhibiting resistance to the NS5B anchor drug SOF.

Our findings of high RAS prevalence among persons with virological failure following initial DAA treatment are consistent with previous reports from clinical trials [31].

A pooled analysis of RAS data from phase III clinical trials (ASTRAL-1–3, ASTRAL-5, and POLARIS-2–3) in patients with SOF/VEL failure reported a RAS prevalence of 93%–100% across GT 1–6 with no SOF resistance [31]. Similarly, we observed minimal differences in RAS prevalence between genotypes, although the sample size of pooled analyses for some GTs was low. Data were mainly available for GT1, GT3, GT4, and GT6 with RAS prevalence ranging from 68% for GT3 and 100% for GT6. Our findings are also consistent with data from the ALLY-3 phase III clinical trial in patients with SOF/DAC failure where a high prevalence of NS5A inhibitor RAS and a low prevalence of NS5B RAS were reported [32]. The low prevalence of SOF resistance in persons with virological failure following treatment is attributed to its high genetic barrier and the impact of NS5B mutations on viral fitness, which also explains the rapid reversion of NS5B RAS upon cessation of treatment [33, 34]. In contrast, NS5A inhibitor RAS have been shown to persist for more than 5 years after treatment cessation due to low fitness cost to the virus [35, 36], which may negatively impact retreatment with the same regimen [37] as well as increase the risk of possible onward transmission of drug-resistant virus [15–17]. The high level of RAS to NS5A inhibitors may also be due to the presence of clinically relevant polymorphisms which may exceed 10% in some genotypes [38].

We estimated absolute number of potential treatment failures and those with RAS to specific drug classes, assuming cure rates of 94% and 91% reported from meta-analyses or large studies for SOF/VEL [4] and SOF/DAC [39], respectively, and if one million people are treated with these DAA regimens annually. The absolute number expected to not achieve SVR with DAA therapy would be between 60 000 and 90 000 people of which between 47 000 and 73 000 would have drug resistance mutations predominantly to the NS5A inhibitor drug class, but only 1000 would have resistance to SOF. Overall, the high prevalence of NS5A-RAS reported among persons with virological failure after initial treatment with pan-genotypic DAA regimens raises uncertainties for retreatment with the same regimen or with an NS5A inhibitor containing dual regimen and hence multidrug resistance [37, 40].

The observed level of resistance to GLE and PIB is also consistent with previous reports [41]. GLE has a higher genetic barrier to the selection of RAS, which is reflected in the lower prevalence of NS3/4A PI RAS observed in this review. Overall, given the high cure rates associated with GLE/PIB (97%), it would be expected that comparatively few of the patients with GLE/PIB virological failure (estimated at about 23 400 patients per one million patients) will harbor RAS compared to other DAAs.

High cure rates have also been reported for the widely adopted retreatment—96.4% for GLE/PIB and 88% for the triple combination SOF/VEL/VOX [42, 43]. However, these regimens are not yet available as generic products and there is therefore limited access in LMICs. Despite the high SVR rates with retreatment, a high proportion of the few who fail to achieve cure will have RAS. As with those failing initial DAA treatment, levels of drug resistance during retreatment are mainly driven by RAS to NS5A inhibitors. Among those with virological failure following retreatment with GLE/PIB, nearly 1 in every 2 had RAS to the NS3/4A PI GLE. Based on the assumption that 4%–12% of people retreated with GLE/PIB or SOF/VEL/VOX, respectively may not be cured, it is estimated that 4000–10 800 of 100 000 individuals retreated with these regimens would have RAS. Our analyses however did not differentiate between resistance emerging during initial treatment failure and after retreatment; therefore, we cannot ascertain the proportion of RAS that is directly attributable to the retreatment regimens. Overall, there is need for further data to guide retreatment strategies for these patients that may include extending the duration of therapy and/or use of ribavirin or in the case of patients initially failing GLE/PIB retreatment, the addition of SOF as was observed in MAGELLAN-3 trial [44].

Expanded access to testing and treatment will be key to achieve the 2030 HCV elimination targets of 90% testing and diagnosis coverage of those infected and 80% treatment coverage of those diagnosed. This should be complemented by strategies to prevent selection of RAS through use of DAA regimens with a high genetic barrier to the selection of drug resistance, especially in populations with hard-to-treat non-epidemic genotypes, such as GT 1l, 3b, 3g, 4r, 6u, and 6v, promotion of optimal adherence, establishing the presence of cirrhosis using non-invasive tests such as APRI score or transient elastography to guide appropriate treatment duration, the use of ribavirin as well as routine monitoring of SVR rates and population-level sentinel surveillance of RAS [2, 3].

Our analysis has important limitations. First, our review only included studies that had access to and reported data on drug resistance and included only publications in English language and studies from certain regions would therefore not be represented. As a result, we did not evaluate for regional variations, and the assessment of RAS by genotype was limited by small numbers with some of rarer genotypes in the pooled analyses. The lack of data on RAS and of overall treatment efficacy with respect to non-epidemic genotypes (those with a limited distribution, eg, 4r and 3b as compared to epidemic, which have a global distribution) may have implications for treatment outcomes as DAAs are rolled out in regions where non-epidemic genotypes predominate [45–47]. Comparatively lower efficacy has been observed in 2 available trials involving the use of first-generation NS5A containing DAAs in regions where GT3b and GT4r are prevalent [48]. Similarly, studies conducted in migrant communities in the global north have shown sub-optimal cure rates for people infected with non-epidemic genotypes [49–52]. The lower SVR rate with non-epidemic genotypes treated with NS5A-based DAA has been attributed to intrinsic high levels of clinically relevant polymorphisms in these genotypes [53]. Overall, our review and other studies highlight the need for more empirical data, complemented by modelling studies on SVR rates and the prevalence of RAS in regions where non-epidemic genotypes predominate as DAA treatment becomes more widely available in these settings [6, 46].

Some studies have shown that SVR can be achieved in some patients despite the presence of RAS [18–20], indicating that that the clinical relevance of the high prevalence of RAS observed in this and other analyses may be overestimated. There is a need for further studies to differentiate between virological failure attributable to RAS versus other factors to inform management strategies, which may include adherence support, retreatment with newer DAAs, extending the duration of treatment with same regimen, or the addition of ribavirin. There is also a need to determine the role of subtypes in retreatment strategies.

Finally, the high degree of heterogeneity observed in this analysis due to low sample sizes for certain genotypes can be addressed through ongoing strategies to promote pooling of data to provide more precise estimates of RAS prevalence. Opportunities to contribute data to pooled databases, such as the SHARED database [14, 54], will enable additional analyses on the patterns and corelates of RAS globally and across different genotypes. Moreover, there is a need to establish simple, sentinel surveillance to determine the prevalence and patterns of RAS, especially in Africa where endemic/non-epidemic genotypes are common and for which information on RAS is very limited.

In summary, our analyses show that resistance associated mutations are common among the small proportion of patients with virological failure following initial or re-treatment using WHO-recommended DAAs. The findings of this review highlight the need for surveillance of DAA-associated resistance, to assess risk factors for their development and clinical impact to inform best practice strategies for re-treatment.

## Supplementary Data

Supplementary materials are available at *Clinical Infectious Diseases* online. Consisting of data provided by the authors to benefit the reader, the posted materials are not copyedited and are the sole responsibility of the authors, so questions or comments should be addressed to the corresponding author.
