# Supplementary material for: Prevalence of Drug Resistance Associated Substitutions in Persons With Chronic Hepatitis C Infection and Virological Failure Following Initial or Re-treatment With Pan-genotypic Direct-Acting Antivirals: A Systematic Review and Meta-analysis
Source: Clin Infect Dis. 2024 Oct 3;79(6):1437–46. doi: 10.1093/cid/ciae431 (PMC11650865; doi:10.1093/cid/ciae431)
Supplement: ciae431_Supplementary_Data [file ciae431_supplementary_data.docx]

Article type: original article

Short title: Prevalence of drug resistance to direct acting agents for HCV treatment

Title: Prevalence of drug resistance associated substitutions in hepatitis C-infected patients whose WHO-recommended direct acting agents for initial treatment and retreatment fails: a systematic review and meta-analysis

Seth Inzaule^1^, Phillippa Easterbrook^2^, Ashley Latona^3^, Nathan P. Ford^2^, William Irving^4^, Philippa C. Matthews^5^, Marco Vitoria^2^, Chris Duncombe^6^, Amalia Giron^7^, Suzanne McCluskey^8^, Olufunmilayo Lesi^2^, Serge Tchamgoue^9^, Rachel Halford^10^, Danjuma Adda^10^, Emma Thomson^11^, Geoff Dusheiko^12^, Michael R. Jordan^3^

1. Amsterdam Institute for Global Health and Development, and Department of Global Health, Amsterdam UMC, University of Amsterdam, Amsterdam, The Netherlands

2. HIV, Hepatitis and Sexually Transmitted Infection Department, World Health Organization, Geneva, Switzerland

3. Tufts University School of Medicine, Boston, MA, USA

4. School of Life Sciences, Division of Microbiology and Infectious Diseases, The University of Nottingham, Nottingham, England

5. The Francis Crick Institute, London, England

6. International Association of Providers of AIDS Care, Washington, DC, USA

7. Independent Consultant, Guatemala

8. Massachusetts General Hospital, Boston, USA

9. Faculty of Medicine and Pharmaceutical Sciences, University of Douala, Douala, Cameroon

10. World Hepatitis Alliance, Geneva, Switzerland

11. University of Glasgow, Glasgow, Scotland

12. Institute for Global Health, University College London, London, England

Table of Contents

[Search terms (or equivalent): 3](#_Toc163902449)

[Overview of data extraction 4](#_Toc163902450)

[Modified Johanna Briggs institute critical appraisal tool for systematic reviews of prevalence studies^1^ 4](#_Toc163902451)

[Table S1: Characteristics of studies of patients in whom Sofosbuvir/Velpatasvir first-line HCV treatment is failing 6](#_Toc163902452)

[Table S2: Characteristics of studies of patients in whom Sofosbuvir/Daclatasvir first-line HCV treatment is failing 8](#_Toc163902453)

[Table S3: Characteristics of studies of in whom Glecaprevir/Pibrentasvir first-line HCV treatment is failing 11](#_Toc163902454)

[Table S4: Characteristics of studies of patients in whom Glecaprevir/Pibrentasvir second-line HCV treatment is failing 12](#_Toc163902455)

[Table S6: Quality of evidence for studies on resistance among HCV patients treated with sofosbuvir+velpasavir first-line direct acting agents 16](#_Toc163902456)

[Table S7: Quality of evidence for studies on resistance among HCV patients treated with sofosbuvir+daclatasvir first-line direct acting agents 18](#_Toc163902457)

[Table S8: Quality of evidence for studies on resistance among HCV patients treated with glecaprevir+pibrentasvir first-line direct acting agents 20](#_Toc163902458)

[Table S9: Quality of evidence for studies on resistance among HCV patients treated with glecaprevir+pibrentasvir second-line direct acting agents 21](#_Toc163902459)

[Table S10: Quality of evidence for studies on resistance among HCV patients treated with sofosbuvir+velpasavir +voxilaprevir second-line direct acting agents 22](#_Toc163902460)

[Table S11: Distribution of included studies for RAS prevalence among those in whom WHO-recommended first-line regimens are failing, by WHO region 23](#_Toc163902461)

[Table S12: Distribution of included studies for RAS prevalence among in whom WHO-recommended first-line regimen are failing, by type of genotype 24](#_Toc163902462)

[Table S13: Distribution of included studies for RAS prevalence among those in whom WHO-recommended second-line regimens are failing, by WHO region 24](#_Toc163902463)

[Table S14: Distribution of included studies for RAS prevalence among those in whom WHO-recommended second-line regimens are failing, by type of genotype 24](#_Toc163902464)

[Table S15: Mutation patterns among patients in whom first-line antiviral containing sofosbuvir and velpatasvir failed 25](#_Toc163902465)

[Table S16: Mutation patterns among patients in whom first-line antiviral containing sofosbuvir and daclatasvir failed 25](#_Toc163902466)

[Table S17: Mutation patterns among patients in whom first-line antiviral containing glecaprevir and pibrentasvir failed 26](#_Toc163902467)

[Table S18: Mutation patterns among patients in whom second-line antiviral containing glecaprevir and pibrentasvir failed 27](#_Toc163902468)

[Table S19: Mutation patterns among patients in whom second-line antiviral containing sofosbuvir/velpasavir /voxilaprevir failed 28](#_Toc163902469)

Search strategy

We used search terms related to HCV infection, DAA, and RAS (appendix pp 1) to identify studies published between January 2014 and July 2023. The start date restrictions correspond to the year when pangenotypic DAAs were first approved by the United States Federal Drug Administration (FDA) for clinical use. We used separate search syntax for studies assessing initial treatment and retreatment (below). We also searched the reference lists of the included studies and review articles published during the defined period, and abstracts presented at key HCV conferences including the European Association for the Study of the Liver (EASL), International Liver Congress, Conference on Retroviruses and Opportunistic Infections (CROI), American Association for the Study of Liver Diseases (AASLD) and the World Hepatitis Summit from the same period.

# Search terms (or equivalent):

Search strategy 1: Prevalence and patterns of RAS among patients failing first-line DAAs

((((HCV Infections[MeSH] OR hepatitis C virus [MeSH] OR HCV[tiab] OR viral hepatitis c*[tiab] OR hcv[tiab] OR HCV infect*[tiab] OR hepatitis C virus[tiab] AND (((direct-acting antivirals [Title/Abstract]) OR (DAA[Title/Abstract])) OR (interferon-free treatment[Title/Abstract]))) AND (((Resistant Associated Mutations[Title/Abstract]) OR (RAS [Title/Abstract])) OR ((mutations[MeSH]))) AND (2014:2023[pdat]))))

Search strategy 2: Prevalence and patterns of RAS among patients failing retreatment DAA

((((HCV Infections[MeSH] OR hepatitis C virus [MeSH] OR HCV[tiab] OR viral hepatitis c*[tiab] OR hcv[tiab] OR HCV infect*[tiab] OR hepatitis C virus[tiab] AND (((direct-acting antivirals [Title/Abstract]) OR (DAA[Title/Abstract])) OR (interferon-free treatment[Title/Abstract]))) AND ((re-treatment[Title/Abstract]) OR (retreatment)) AND (((Resistant Associated Mutations[Title/Abstract]) OR (RAS [Title/Abstract])) OR ((mutations[MeSH]))) AND (2014:2023[pdat]))))

# **Overview of data extraction**

Data were extracted from included studies using Microsoft Excel 2023 for Mac. We extracted the following variables:

*Study characteristics*

- first author and year of publication, if published
- study years; location (i.e., country and WHO region)
- study design
- treatment history

*Outcomes*

- Proportion of patients with sustained virological response if available
- Genotype information
- number of patients with treatment failure or relapse
  - and by genotype
- number of patients with resistance associated substitutions during treatment failure
  - and by genotype

# **Modified Johanna Briggs institute critical appraisal tool for systematic reviews of prevalence studies^1^**

Assessment of methodological quality of included studies was done using a modified Joanna Briggs Institute (JBI) critical appraisal tool^1^. The tool has nine domains, and the responses are graded into three options i) yes if the item assessed was performed appropriately and sufficient information about its performance is provided in the manuscript ii) no, if the item was not performed appropriately; and “unclear” if sufficient information is not available in the article to provide sufficient information to be able to determine whether the item was performed appropriately or not. The nine questions and modified queries are indicated below:

1. Was the sample frame appropriate to address the target population?
2. Were study participants sampled in an appropriate way?
3. Was the sample size adequate?
4. Were the study subjects and the setting described in detail?
5. Was the data analysis conducted with sufficient coverage of the identified sample?
6. Were valid methods used for the identification of the condition?
7. Was the condition measured in a standard, reliable way for all participants?
8. Was there appropriate statistical analysis?
9. Was the response rate adequate, and if not, was the low response rate managed appropriately?

Answers: Yes/ No/ Unclear/ Not applicable

The specific questions were considered as follows:

1. Appropriateness of the sample frame for the target population: this item considered the overall strategy for selection of potential participants, the characteristics of the population of patients either treated with HCV, or those failing DAA treatment in the respective setting, and the appropriateness of eligibility criteria for inclusion.
2. Appropriateness of participant sampling: this item considered if participants were included consecutively or randomly into the study or if a convenience sample was used. Studies that included all patients failing treatment were considered a census. Equally studies that collected data from multiple sites for patients failing treatment was considered a census if the description provided sufficient detail to judge if all samples from patients failing treatment were included in the analysis.
3. Description of participants and setting was considered appropriate if sufficient detail was provided. Includes description of vital information such as country, first or second-line treatment.
4. Sample size criterion was not included in the assessment as evaluation of resistance is a secondary outcome to sustained virological response, and the overall population with treatment failure is usually very small.
5. Analysis conducted with sufficient coverage: this item considered whether resistance testing and/or reporting differed between subgroups of study participants e.g., those with advanced liver disease or given genotypes.
6. Valid methods used for the identification of the condition: this item evaluated if resistance testing was done using genotyping or phenotyping methods and if for all target genes and covering all known mutations.
7. Condition measured in a standard, reliable way for all participants: this item required to report that the same strategy for ascertaining DAA resistance among those failing treatment was used in all participants.
8. Appropriateness of statistical analysis: this item evaluated if there was a clear identification of numerator and denominator to allow for appropriate analyses.
9. Adequate response rate: this item assessed if the study reported on refusals to participate into the study, unnecessary or high number of exclusions from resistance testing among failures and genotyping failure or missingness of results. If exclusions and missingness were not discussed the information was graded as “unclear.” Nonetheless some missingness/ exclusions were considered unavoidable for example some level of sequencing failure. Studies with missing information on key aspects were graded as “no” (response rate inadequate) or “unclear” (missingness not described).

Studies were considered as having a low risk of bias if missing information from at most two domains, moderate for three domains and high risk if missing information from four or more domains.

# **Table S1:** Characteristics of studies of patients in whom Sofosbuvir/Velpatasvir first-line HCV treatment is failing

| Study | Study name | Year | WHO region | Country | Study design | N | SVR** | N failing/relapse | GT1 | GT2 | GT3 | GT4 | GT5 | GT6 |
| --- | --- | --- | --- | --- | --- | --- | --- | --- | --- | --- | --- | --- | --- | --- |
| Quinn N et al^2^ | - | 2023 | EURO | Ireland | Retrospective analysis of patients failing treatment (1 clinic) |  |  | 3 | 3 |  |  |  |  |  |
| Xie Z et al^3^ | - | 2022 | SEARO | China | Retrospective analysis of patients failing treatment from a cohort study | 98 | 96% | 3 | 1 |  | 1 |  |  | 1 |
| Mushtaq S et al^4^ |  | 2022 | EMRO | Pakistan | Retrospective analysis of patients failing treatment from a cohort study | - | - | 22 |  |  | 22 |  |  |  |
| Onofrio et al^5^ | Canadian Network Undertaking against HCV (CANUHC) | 2021 | AMRO | Canada | Baseline data of patients being retreated after DAA failure in a prospective multi-center real-world cohort study (17 sites) | - | - | 18 |  |  |  |  |  |  |
| Wang P et al^6^ | HCV-TARGET Network | 2021 | AMRO | United States | Baseline data of patients being retreated in an open-label trial | 177 | 91.5% | 9 | 9 |  |  |  |  |  |
| Jia Y et al^7^ |  | 2021 | SEARO | China | Retrospective analysis of patients infected with GT6 failing treatment | 8 | 88% | 1 |  |  |  |  |  | 1 |
| Di Maio VC et al^8^ | Vironet-C study | 2021 | EURO | Italy | Multi-country cohort study of patients failing DAA | 47 | 96% | 15 |  |  | 15 |  |  |  |
| Isfordink CJ et al^9^ | - | 2021 | EURO | Netherlands | Retrospective analysis of patients with non-epidemic genotype failing treatment from a nationwide cohort study | 24 | 96% | 1 |  |  | 1 |  |  |  |
| Di Stefano M et al^10^ | - | 2021 | EURO | Italy | Retrospective analysis of patients failing treatment or relapse referred to a referral site | - | - | 3 |  |  |  |  |  |  |
| Fourati S et al^11^ | - | 2020 | EURO | France | Collection of patients infected with GT3a failing treatment | - | - | 2 |  |  | 2 |  |  |  |
| Dietz J et al^12^ | - | 2020 | EURO | Germany | Retrospective analysis of patients infected with GT4 failing treatment | - | - | 3 |  |  |  | 3 |  |  |
| Douglas M et al^13^ | - | 2020 | WPRO | Australia | Retrospective analysis of patients failing treatment from a nationwide cohort study | - | - | 52 | 8 | 3 | 38 |  |  | 3 |
| Alessio L et al^14^ | - | 2020 | EURO | Italy | Retrospective analysis of HIV co-infected patients failing treatment | - | - | 1 | 1 |  |  |  |  |  |
| Fourati S et al^15^ | - | 2019 | EURO | France | Collection of patients infected with GT4r failing treatment | - | - | 1 |  |  |  | 1 |  |  |
| Gozlan Y et al^16^ | - | 2019 | EMRO | Israel | Collection of patients failing treatment (12 sites) | - | - | 1 |  |  | 1 |  |  |  |
| Di Maio VC et al^17^ | - | 2021 | EURO | Italy | Retrospective analysis of patients failing treatment | - | - | 58 | 20 | 10 | 26 | 2 |  |  |
| Dietz J et al^18^ | European HCV Resistance Study Group | 2019 | EURO | - | Retrospective analysis of patients infected with GT3 failing treatment in the European resistance database |  |  | 60 |  |  | 60 |  |  |  |

** Excludes those who relapsed after achieving initial SVR at end of treatment course

# **Table S2:** Characteristics of studies of patients in whom Sofosbuvir/Daclatasvir first-line HCV treatment is failing

| Study | Study name | Year | WHO region | Country | Study design | N | SVR | N failing/relapse | GT1 | GT2 | GT3 | GT4 | GT5 | GT6 |
| --- | --- | --- | --- | --- | --- | --- | --- | --- | --- | --- | --- | --- | --- | --- |
| Rodrigues JPV et al^19^ | - | 2023 | AMRO | Brazil | Analysis of patients failing first-line treatment from a cohort study in a tertiary hospital | 105 | 88% | 6 |  |  | 6 |  |  |  |
| Quinn N et al^2^ | - | 2023 | EURO | Ireland | Retrospective analysis of patients failing treatment (1 clinic) |  |  | 4 | 1 |  | 3 |  |  |  |
| Mushtaq et al^4^ |  | 2022 | EMRO | Pakistan | Retrospective analysis of patients infected with GT3 failing treatment from a cohort study | - | - | 54 |  |  | 54 |  |  |  |
| Wang P et al^6^ | HCV-TARGET Network | 2021 | AMRO | United States | Baseline data of patients being retreated in an open-label trial | 177 | 91.5 | 1 | 1 |  |  |  |  |  |
| Elhence A et al^20^ |  | 2021 | SEARO | India | Baseline data of patients being retreated in a tertiary referral site | 17 |  | 15 | 3 |  | 12 |  |  |  |
| Di Maio VC et al^8^ | Vironet-C study | 2021 | EURO | Italy | Cohort study of patients with GT 3 failing DAA | 47 | 95.7% | 91 |  |  | 91 |  |  |  |
| Isfordink CJ et al^9^ | - | 2021 | EURO | Netherlands | Retrospective analysis of patients with non-epidemic genotype failing treatment from a nationwide cohort study | 31 | 90.0% | 3 |  | 1 | 2 |  |  |  |
| Campos GRF et al^21^ | - | 2021 | AMRO | Brazil | Retrospective analysis of patients infected with GT3 failing treatment | 82 | 92.7% | 6 |  |  | 6 |  |  |  |
| Ruiz I et al^22^ | - | 2018 | EURO | France | Retrospective analysis of patients with inherited blood disorders failing treatment | 12 | 91.6% | 1 |  | 1 |  |  |  | 1 |
| Di Stefano M et al^10^ | - | 2021 | EURO | Italy | Retrospective analysis of patients failing treatment or relapse referred to a referral site | - | - | 13 |  |  |  |  |  |  |
| Fourati S et al^11^ | - | 2020 | EURO | France | Collection of patients infected with GT3a failing treatment | - | - | 6 |  |  | 6 |  |  |  |
| Dietz J et al^12^ | - | 2020 | EURO | Germany | Retrospective analysis of patients failing treatment | - | - | 4 |  |  |  | 4 |  |  |
| Douglas M et al^13^ | - | 2020 | WPRO | Australia | Retrospective analysis of patients failing treatment from a nationwide cohort study | - | - | 277 | 18 | 1 | 257 |  |  | 1 |
| Fedorchenko S et al^23^ | - | 2020 | EURO | Ukraine | Baseline data of patients infected with GT1b being retreated in a referral site | - | - | 2 | 2 |  |  |  |  |  |
| Chen Q et al^24^ | - | 2020 | EURO | Spain | Retrospective analysis of patients failing treatment from 39 clinics nationwide | - | - | 35 | 8 |  | 27 |  |  |  |
| Gozlan Y et al^16^ | - | 2019 | EMRO | Israel | Collection of patients failing treatment (12 sites) | - | - | 5 | 1 |  | 4 |  |  |  |
| Minosse C et al^25^ | - | 2018 | EURO | Italy | Retrospective analysis of patients infected with GT4 failing treatment | 5 | 80% | 1 |  |  |  | 1 |  |  |
| Dietz J et al^26^ | European HCV Resistance Study Group | 2020 | EURO | Austria, Germany, Poland, Spain, Switzerland | Retrospective analysis of patients failing treatment in the European resistance multi-country study | - | - | 82 | 29 |  | 50 | 3 |  |  |
| Fourati S et al^15^ | - | 2019 | EURO | France | Collection of patients infected with GT4r failing treatment |  |  | 2 |  |  |  | 2 |  |  |
| Dietz J et al^18^ | European HCV Resistance Study Group | 2019 | EURO |  |  | - | - | 124 |  |  | 124 |  |  |  |
| Alessio L et al^14^ | - | 2020 | EURO | Italy | Retrospective analysis of HIV co-infected patients failing treatment | - | - | 2 | 1 |  | 1 |  |  |  |
| Bachofner J et al^27^ | - | 2018 | EURO | Switzerland | Retrospective analysis of patients failing treatment at 3 tertiary clinics | 76 | 97.3% | 2 |  |  | 2 |  |  |  |
| Salmon D. et al^28^ | French ANRS CO13 HEPAVIH | 2018 | EURO | France | Retrospective analysis of HIV co-infected patients failing treatment | 240 | 96.3% | 6 | 5 |  |  | 1 |  |  |
| Paolucci et al^29^ | - | 2017 | EURO | Italy | Retrospective analysis of patients failing treatment in a referral site | - | - | 4 | 3 |  | 1 |  |  |  |
| Di Maio VC et al^17^ | - | 2017 | EURO | Italy, Spain, Germany | Collection of patients failing treatment | - | - | 4 | 2 |  | 2 |  |  |  |

** Excludes those who relapsed after achieving initial SVR at end of treatment course

# **Table S3:** Characteristics of studies of in whom Glecaprevir/Pibrentasvir first-line HCV treatment is failing

| Study | Study name | Year | WHO region | Country | Study design | N | SVR | N failing/relapse | GT1 | GT2 | GT3 | GT4 | GT5 | GT6 |
| --- | --- | --- | --- | --- | --- | --- | --- | --- | --- | --- | --- | --- | --- | --- |
| Quinn N et al^2^ | - | 2023 | EURO | Ireland | Retrospective analysis of patients failing treatment (1 clinic) |  |  | 2 | 1 |  | 1 |  |  |  |
| Xie Z et al^3^ | - | 2022 | SEARO | China | Retrospective analysis of patients failing treatment from a cohort study | 13 | 85% | 2 |  |  | 2 |  |  |  |
| Dietz J et al^30^ | European Resistance Study Group | 2022 | EURO | Austria, Belgium, Germany and Switzerland | Retrospective analysis of patients infected with GT1 and Gt3 failing treatment from a multi-country study |  |  | 31 |  |  | 31 |  |  |  |
| Di Maio VC et al^8^ | Vironet-C study | 2021 | EURO | Italy | Cohort study of patients with GT 3 failing DAA | 30 | 97% | 5 |  |  | 5 |  |  |  |
| Di Stefano M et al^10^ |  | 2021 | EURO | Italy | Retrospective analysis of patients failing treatment or relapse referred to a referral site |  |  | 1 |  |  |  |  |  |  |
| Douglas M et al^13^ |  | 2020 | WPRO | Australia | Retrospective analysis of patients failing treatment from a nationwide cohort study |  |  | 6 | 2 |  | 4 |  |  |  |
| Alessio L et al^14^ |  | 2020 | EURO | Italy | Retrospective analysis of HIV co-infected patients failing treatment |  |  | 1 | 1 |  |  |  |  |  |
| D’Ambrosio R et al^31^ | Italy (NAVIGATORE-Lombardia Network) | 2019 | EURO | Italy | Retrospective analysis of patients failing treatment from a large cohort study | 680 | 99,3 | 5 |  | 3 | 2 |  |  |  |
| De Salazar A et al^32^ |  | 2020 | EURO | Austria, Germany, Italy, Spain and Switzerland | Retrospective analysis of patients failing treatment from a multi-country study | 52 | 97.8% | 1 |  |  | 1 |  |  |  |
| Pearlman B et al^33^ |  | 2019 | AMRO | US | Baseline data of patients infected with GT1 and GT3 being retreated in a tertiary referral site | - |  | 31 |  |  |  |  |  |  |
| Di Maio VC et al^17^ |  | 2019 | EURO | Italy, Spain, Germany | Multi-country cohort study of patients failing DAA |  |  | 43 | 11 | 19 | 12 | 1 |  |  |
| Dietz J et al^18^ | European HCV Resistance Study Group | 2019 | EURO | - |  |  |  | 12 |  |  | 12 |  |  |  |
| Flamm S et al^34^ | Surveyor-2, Endurance III, Magellan 2, Expedition 2 | 2018 |  |  | Retrospective analysis of patients infected with GT3 failing treatment in phase II/III clinical trials | 693 | 95% | 19 |  |  | 19 |  |  |  |

** Excludes those who relapsed after achieving initial SVR at end of treatment course

# **Table S4:** Characteristics of studies of patients in whom Glecaprevir/Pibrentasvir second-line HCV treatment is failing

| Study | Study | Year | WHO region | Country | Study design | N | SVR | N failing/relapse | GT1 | GT2 | GT3 | GT4 | GT5 | GT6 |
| --- | --- | --- | --- | --- | --- | --- | --- | --- | --- | --- | --- | --- | --- | --- |
| Quinn N et al^2^ | - | 2023 | EURO | Ireland | Retrospective analysis of patients failing treatment (1 clinic) |  |  | 1 | 1 |  |  |  |  |  |
| Poordad F et al^35^ | MAGELLAN-1 part 2 | 2018 | AMRO, EURO, WPRO | Australia, France, New Zealand, Puerto Rico, Spain, United Kingdom, United States | Analysis of patients failing second-line treatment infected with GT1 and 4 from a multi-center, randomized, open-Label trial | 91 | 90% | 9 | 9 |  |  |  |  |  |
| Lok AS et al^36^ | US,HCV-TARGET Network | 2019 | EURO | US | Analysis of patients failing second-line treatment from a multicenter phase 3b open label trial | 177 | 92.7% | 14 | 14 |  |  |  |  |  |
| Mawatari S et al^37^ |  | 2020 | WPRO | Japan | Analysis of a cohort of patients infected with GT1 failing second-line treatment | 24 | 87.5% | 3 | 3 |  |  |  |  |  |
| Uemura H et al^38^ |  | 2019 | WPRO | Japan | Analysis of a cohort of patients infected with GT1,2,3 failing second-line treatment | 42 | 92.9% | 3 | 3 |  |  |  |  |  |
| Sezaki H et al^39^ |  | 2019 | WPRO | Japan | Analysis of a cohort of patients infected with GT1,2,3 failing second-line treatment | 88 | 97% | 2 | 2 |  |  |  |  |  |
| Osawa M et al^40^ |  | 2019 | WPRO | Japan | Analysis of a cohort of patients infected with GT1,2,3 failing second-line treatment | 30 | 93.3% | 2 | 2 |  |  |  |  |  |

** Excludes those who relapsed after achieving initial SVR at end of treatment course

**Table S5:** Characteristics of studies of patients in whom Sofosbuvir/Velpatasvir/Voxilepravir second-line HCV treatment is failing

| Study | Study | Year | WHO region | Country | Study design | N | SVR | N failing/relapse | GT1 | GT2 | GT3 | GT4 | GT5 | GT6 |
| --- | --- | --- | --- | --- | --- | --- | --- | --- | --- | --- | --- | --- | --- | --- |
| Quinn N et al^2^ | - | 2023 | EURO | Ireland | Retrospective analysis of patients failing treatment (1 clinic) |  |  | 2 | 2 |  |  |  |  |  |
| Smith DA et al^41^ | HCV Research UK and other UK data | 2021 | EURO | United Kingdom | Analysis of a cohort of patients failing second-line treatment | 144 | 91% | 15 | 2 |  | 12 | 1 |  |  |
| Garcia-Cehic D et al^42^ |  | 2021 | EURO |  | Analysis of patients failing second-line treatment | - | - | 5 | 2 |  | 3 |  |  |  |
| Dietz J et al^43^ | European Resistance Study Group | 2021 | EURO |  | Retrospective analysis of patients failing second-line treatment in the European Resistance Study |  |  | 39 | 19 |  | 18 |  |  |  |
| De Salazar A et al^32^ | Vironet-C | 2020 | EURO | Italy | Retrospective analysis of patients failing treatment | 52 | 97.8% | 1 |  |  | 1 |  |  |  |
| Degasperi E et al^44^ | NAVIGA- TORE Lombardia and Veneto Networks | 2019 | EURO | Italy | Retrospective analysis of a cohort of patients failing second-line treatment | 179 | 91% | 7 | 3 |  | 3 | 1 |  |  |
| Eleanor W et al^45^ | RESOLVE | 2019 | AMRO | US | Analysis of patients infected with GT1 failing second-line treatment from a multi-center, randomized trial | 77 | 91% | 4 | 4 |  |  |  |  |  |
| Pearlman B et al^33^ |  | 2019 | AMRO | US | Retrospective analysis of patients failing second-line treatment | 31 | 90% | 2 | 1 |  |  |  |  |  |
| Di Maio VC et al^17^ | Vironet-C study (cohort) | 2019 | EURO | Italy | Retrospective analysis of patients failing treatment | 83 | 94% | 5 | 4 |  |  | 1 |  |  |
| Sarrazin C et al^46^ | POLARIS 1 &4 | 2018 |  |  | Analysis of patients infected with GT1, 2 or 3 failing second-line treatment from a multi-center, randomized trial | 248 | 97.1% | 8 | 3 |  | 4 | 1 |  |  |
| Bourlière M et al^47^ | Polaris 1 Open-label |  | AMRO/EURO/SEARO | SA, France, Canada, the UK, Germany, Australia, and New Zealand | Analysis of patients infected with GT1 failing second-line treatment from a multi-center, randomized trial | 147 | 97% | 4 | 4 |  |  |  |  |  |
| Lawitz et al^48^ | - | 2017 | AMRO |  | Analysis of patients infected with GT1 failing second-line treatment from a multi-center, randomized trial | 48 | 98% | 1 | 1 |  |  |  |  |  |
| Gozlan Y et al^49^ | - | 2020 | EMRO | Israel | Analysis of patients failing DAA nationwide | - | - | 1 |  |  | 1 |  |  |  |

** Excludes those who relapsed after achieving initial SVR at end of treatment course

# **Table S6:** Quality of evidence for studies on resistance among HCV patients treated with sofosbuvir+velpasavir first-line direct acting agents

| Study | Representativeness of target population | Appropriate sampling | Participant and setting description | Sample size | Completeness in ascertainment of outcome in all groups | Appropriate method for ascertainment of outcome | Appropriate method for ascertainment of outcome done in all groups | Appropriate statistical analyses | Adequate response rate/non-missingness |
| --- | --- | --- | --- | --- | --- | --- | --- | --- | --- |
| Quinn N et al^2^ | Yes | Yes | Yes | Not assessed | Unclear | Unclear | Unclear | Yes | Yes |
| Xie Z et al^3^ | Yes | Yes | Yes | Not assessed | Yes | Yes | Yes | Yes | Yes |
| Mushtaq S et al^4^ | Yes | Yes | Yes | Not assessed | Yes | Yes | Yes | Yes | Yes |
| Onofrio et al^5^ | Unclear | Unclear | Yes | Not assessed | Unclear | Yes | Yes | Yes | Unclear |
| Wang P et al^6^ | Yes | Yes | Yes | Not assessed | Unclear | No* | No* | Yes | Yes |
| Jia Y et al^7^ | Yes | Yes | Yes | Not assessed | Yes | Yes | Yes | Yes | Yes |
| Di Maio VC et al^8^ | Yes | Yes | Yes | Not assessed | Yes | Yes | Yes | Yes | Yes |
| Isfordink CJ et al^9^ | Yes | Yes | Yes | Not assessed | Yes | Yes | Yes | Yes | Yes |
| Di Stefano M et al^10^ | Yes | Yes | Yes | Not assessed | Yes | Yes | Yes | Yes | Yes |
| Fourati S et al^11^ | Yes | Yes | Yes | Not assessed | Unclear | Yes | Yes | Yes | Unclear |
| Fourati S et al^15^ | Yes | Yes | Yes | Not assessed | Unclear | Yes | Yes | Yes | Yes |
| Dietz J et al^12^ | Yes | Yes | Yes | Not assessed | Unclear | Yes | Yes | Yes | Yes |
| Douglas M et al^13^ | Yes | Yes | Yes | Not assessed | Yes | Yes | Yes | Unclear | Unclear |
| Alessio L et al^14^ | Yes | Yes | Yes | Not assessed | Yes | Unclear | Unclear | Yes | Unclear |
| Gozlan Y et al^16^ | Yes | Yes | Yes | Not assessed | Yes | No* | No* | Yes | Yes |
| Di Maio VC et al^17^ | Yes | Yes | Yes | Not assessed | Yes | Yes | Yes | Yes | Yes |
| Dietz J et al^18^ | Yes | Yes | Yes | Not assessed | Yes | Yes | Yes | Unclear | Yes |

* Did not report NS5B RAS

# **Table S7:** Quality of evidence for studies on resistance among HCV patients treated with sofosbuvir+daclatasvir first-line direct acting agents

| Study | Representativeness of target population | Appropriate sampling | Participant and setting description | Sample size | Completeness in ascertainment of outcome in all groups | Appropriate method for ascertainment of outcome | Appropriate method for ascertainment of outcome done in all groups | Appropriate statistical analyses | Adequate response rate/non-missingness |
| --- | --- | --- | --- | --- | --- | --- | --- | --- | --- |
| Rodrigues JPV et al^19^ | Unclear | Yes | Yes | Not assessed | Yes | Yes | Yes | Yes | Unclear |
| Quinn N et al^2^ | Yes | Yes | Yes | Not assessed | Unclear | Unclear | Unclear | Yes | Yes |
| Mushtaq et al^4^ | Yes | Yes | Yes | Not assessed | Yes | Yes | Yes | Yes | Yes |
| Wang P et al^6^ | Yes | Yes | Yes | Not assessed | Unclear | No* | No* | Yes | Yes |
| Elhence A et al^20^ | Yes | Yes | Yes | Not assessed | Unclear | Yes | Yes | Yes | No |
| Di Maio VC et al^8^ | Yes | Yes | Yes | Not assessed | Yes | Yes | Yes | Yes | Yes |
| Isfordink CJ et al^9^ | Yes | Yes | Yes | Not assessed | Yes | Yes | Yes | Yes | Yes |
| Campos GRF et al^21^ | Yes | Yes | Yes | Not assessed | Yes | No* | No* | Yes | Yes |
| Ruiz I et al^22^ | No | Yes | Yes | Not assessed | Yes | Yes | Yes | Yes | Yes |
| Di Stefano M et al^10^ | Yes | Yes | Yes | Not assessed | Yes | Yes | Yes | Yes | Yes |
| Fourati S et al^11^ | Yes | Yes | Yes | Not assessed | Unclear | Yes | Yes | Yes | Unclear |
| Fourati S et al^15^ | Yes | Yes | Yes | Not assessed | Unclear | Yes | Yes | Yes | Yes |
| Dietz J et al^12^ | Yes | Yes | Yes | Not assessed | Unclear | Yes | Yes | Yes | Yes |
| Douglas M et al^13^ | Yes | Yes | Yes | Not assessed | Yes | Yes | Yes | Unclear | Unclear |
| Fedorchenko S et al^23^ | Yes | Yes | Yes | Not assessed | Yes | Unclear | Unclear | Yes | Yes |
| Chen Q et al^24^ | No | Yes | Yes | Not assessed | Yes | Unclear | Unclear | Yes | Yes |
| Gozlan Y et al^16^ | Yes | Yes | Yes | Not assessed | Yes | Yes | Yes | Yes | Yes |
| Minosse C et al^25^ | Yes | Yes | Yes | Not assessed | Yes | No* | No* | Yes | Yes |
| Dietz J et al^26^ | No | Yes | Yes | Not assessed | Yes | Yes | Yes | Yes | Yes |
| Dietz J et al^12^ | Yes | Yes | Yes | Not assessed | Yes | Yes | Yes | Yes | Yes |
| Bachofner J et al^27^ | Yes | Yes | Yes | Not assessed | Unclear | Unclear | Yes | Yes | Yes |
| Salmon D. et al^28^ | Yes | Yes | Yes | Not assessed | Yes | Yes | Yes | Yes | Unclear |
| Paolucci et al^29^ | Yes | Yes | Yes | Not assessed | Yes | Yes | Yes | Yes | Yes |
| Di Maio VC et al^17^ | Yes | Yes | Yes | Not assessed | Yes | Yes | Yes | Yes | Yes |
| Dietz J et al^18^ | Yes | Yes | Yes | Not assessed | Yes | Yes | Yes | Unclear | Yes |

* Did not report NS5B RAS

# **Table S8:** Quality of evidence for studies on resistance among HCV patients treated with glecaprevir+pibrentasvir first-line direct acting agents

| Study | Representativeness of target population | Appropriate sampling | Participant and setting description | Sample size | Completeness in ascertainment of outcome in all groups | Appropriate method for ascertainment of outcome | Appropriate method for ascertainment of outcome done in all groups | Appropriate statistical analyses | Adequate response rate/non-missingness |
| --- | --- | --- | --- | --- | --- | --- | --- | --- | --- |
| Quinn N et al^2^ | Yes | Yes | Yes | Not assessed | Unclear | Unclear | Unclear | Yes | Yes |
| Xie Z et al^3^ | Yes | Yes | Yes | Not assessed | Yes | Yes | Yes | Yes | Yes |
| Dietz J et al^30^ | Yes | Yes | Yes | Not assessed | Yes | Yes | Yes | Yes | Yes |
| Di Maio VC et al^8^ | Yes | Yes | Yes | Not assessed | Yes | Yes | Yes | Yes | Yes |
| Di Stefano M et al^10^ | Yes | Yes | Yes | Not assessed | Yes | Yes | Yes | Yes | Yes |
| Douglas M et al^13^ | Yes | Yes | Yes | Not assessed | Yes | Yes | Yes | Unclear | Unclear |
| Alessio L et al^14^ | Yes | Yes | Yes | Not assessed | Yes | Unclear | Unclear | Yes | Yes |
| D’Ambrosio R et al^31^ | Yes | Yes | Yes | Not assessed | Yes | Yes | Yes | Yes | Yes |
| De Salazar A et al^32^ | Yes | Yes | Yes | Not assessed | Yes | Yes | Yes | Yes | Yes |
| Pearlman B et al^33^ | Unclear | Yes | Yes | Not assessed | Yes | Yes | Yes | Unclear | Yes |
| Di Maio VC et al^17^ | Yes | Yes | Yes | Not assessed | Yes | Yes | Yes | Yes | Yes |
| Flamm S et al^34^ | Yes | Yes | Yes | Not assessed | Unclear | Unclear | Yes | Yes | Yes |
| Dietz J et al^18^ | Yes | Yes | Yes | Not assessed | Yes | Yes | Yes | Unclear | Yes |

Considering the quality of evidence for the review question, 13/17 studies assessing RAS in SOF/DAC failures had high quality of evidence, 3 moderate, and 1 had low quality of evidence. For SOF/VEL, 23/26 studies had high quality of evidence and two had moderate quality, while all 12 studies for GLE/PIB had high quality of evidence.

# **Table S9:** Quality of evidence for studies on resistance among HCV patients treated with glecaprevir+pibrentasvir second-line direct acting agents

| Study | Representativeness of target population | Appropriate sampling | Participant and setting description | Sample size | Completeness in ascertainment of outcome in all groups | Appropriate method for ascertainment of outcome | Appropriate method for ascertainment of outcome done in all groups | Appropriate statistical analyses | Adequate response rate/non-missingness |
| --- | --- | --- | --- | --- | --- | --- | --- | --- | --- |
| Quinn N et al^2^ | Yes | Yes | Yes | Not assessed | Unclear | Unclear | Unclear | Yes | Yes |
| Poordad F et al^35^ | Yes | Yes | Yes | Not assessed | Yes | Yes | Yes | Yes | Yes |
| Lok AS et al^36^ | Yes | Yes | Yes | Not assessed | Yes | Yes | Yes | Yes | Yes |
| Mawatari S et al^37^ | No | Yes | Yes | Not assessed | Yes | Yes | Yes | Yes | Yes |
| Uemura H et al^38^ | Yes | Yes | Yes | Not assessed | Yes | Yes | Yes | Yes | Yes |
| Sezaki H et al^39^ | Yes | Yes | Yes | Not assessed | Yes | Yes | Yes | Yes | Yes |
| Osawa M et al^40^ | Yes | Yes | Yes | Not assessed | Yes | Yes | Yes | Yes | Yes |

# **Table S10:** Quality of evidence for studies on resistance among HCV patients treated with sofosbuvir+velpasavir +voxilaprevir second-line direct acting agents

| Study | Representativeness of target population | Appropriate sampling | Participant and setting description | Sample size | Completeness in ascertainment of outcome in all groups | Appropriate method for ascertainment of outcome | Appropriate method for ascertainment of outcome done in all groups | Appropriate statistical analyses | Adequate response rate/non-missingness |
| --- | --- | --- | --- | --- | --- | --- | --- | --- | --- |
| Quinn N et al^2^ | Yes | Yes | Yes | Not assessed | Unclear | Unclear | Unclear | Yes | Yes |
| Smith DA et al^41^ | Yes | Yes | Yes | Not assessed | Yes | Yes | Yes | Yes | Yes |
| Garcia-Cehic D et al^42^ | Unclear | Yes | Yes | Not assessed | Yes | Yes | Yes | Yes | Yes |
| Dietz J et al^43^ | Unclear | Yes | Yes | Not assessed | Yes | Yes | Yes | Yes | Yes |
| De Salazar A et al^32^ | Yes | Yes | Yes | Not assessed | Yes | Yes | Yes | Yes | Unclear |
| Degasperi E et al^44^ | Yes | Yes | Yes | Not assessed | Yes | Yes | Yes | Yes | No |
| Eleanor W et al^45^ | Unclear | Yes | Yes | Not assessed | Yes | Yes | Yes | Yes | Yes |
| Pearlman B et al^33^ | Yes | Yes | Yes | Not assessed | Yes | Yes | Yes | Yes | Yes |
| Di Maio VC et al^17^ | Yes | Yes | Yes | Not assessed | Yes | Yes | Yes | Yes | Yes |
| Sarrazin C et al^46^ | Yes | Yes | Yes | Not assessed | Yes | Yes | Yes | Yes | Yes |
| Bourlière M et al^47^ | Yes | Yes | Yes | Not assessed | Yes | Yes | Yes | Yes | Yes |
| Lawitz et al^48^ | Yes | Yes | Yes | Not assessed | Yes | Yes | Yes | Yes | Yes |
| Gozlan Y et al^49^ | Unclear | Yes | Yes | Not assessed | Yes | Unclear | Unclear | Yes | Yes |

Considering the quality of evidence for the review question, 6/7 studies assessing RAS among those failing GLE/PIB retreatment had high quality of evidence and one had moderate quality of evidence while 11/13 studies assessing RAS among patients failing SOF/VEL/VOX retreatment had high quality of evidence while two had moderate quality of evidence.

# **Table S11:** Distribution of included studies for RAS prevalence among those in whom WHO-recommended first-line regimens are failing, by WHO region

| DAA | Studies (N) | WHO region n (%) | | | | | | |
| --- | --- | --- | --- | --- | --- | --- | --- | --- |
|  |  | AFRO | AMRO | EURO | EMRO | SEARO | WPRO | Unknown |
| SOF/VEL | 14 | 0 | 2 (14) | 7 (50) | 2 (14) | 2 (14) | 1 (7) | - |
| SOF/DAC | 21 | 0 | 2 (10 | 15 (71) | 2 (10) | 1 (5) | 1 (5) | - |
| G/P | 11 | 0 | 1 (10) | 7 (64) | 0 | 1 (10) | 1(10) | 1 (10) |

| DAA | Studies (N) | Number of patients | Genotype n (%) | | | | | | | |
| --- | --- | --- | --- | --- | --- | --- | --- | --- | --- | --- |
|  |  |  | GT1 | GT2 | GT3 | GT4 | GT5 | GT6 | GT7 | Unknown |
| SOF/VEL | 14 | 240 | 34 (14) | 10 (4) | 168 (70) | 6 (3) | 0 | 2 (1) | 0 | 20 (8) |
| SOF/DAC | 21 | 751 | 51 (7) | 2 (0.3) | 649 (86) | 10 (1.3) | 0 | 1 (0.1) | 0 | 38 (5.1) |
| G/P | 11 | 243 | 44 (18) | 40 (16) | 126 (52) | 1 (0.4) | 0 | 0 | 0 | 32 (13) |

# **Table S12:** Distribution of included studies for RAS prevalence among in whom WHO-recommended first-line regimen are failing, by type of genotype

# **Table S13:** Distribution of included studies for RAS prevalence among those in whom WHO-recommended second-line regimens are failing, by WHO region

| DAA | Studies (N) | WHO region (%) | | | | | | |
| --- | --- | --- | --- | --- | --- | --- | --- | --- |
|  |  | AFRO | AMRO | EURO | EMRO | SEARO | WPRO | Unknown |
| G/P | 6* | 0 | 3 | 1 | 2 | 2 | 4 | - |
| SOF/VEL/VOX | 21 | 0 | 2 (10 | 15 (71) | 2 (10) | 1 (5) | 1 (5) | - |

* Includes 1 multi-regional study

| DAA | Studies (N) | Number of patients | Genotype n (%) | | | | | | | |
| --- | --- | --- | --- | --- | --- | --- | --- | --- | --- | --- |
|  |  |  | GT1 | GT2 | GT3 | GT4 | GT5 | GT6 | GT7 | Unknown |
| G/P | 7 | 34 | 34 (100) | 0 | 0 | 0 | 0 | 0 | 0 | - |
| SOF/VEL/VOX | 11 | 90 | 41 (46) | 0 | 45 (50) | 4 (4) | 0 | 0 | 0 | - |

# **Table S14:** Distribution of included studies for RAS prevalence among those in whom WHO-recommended second-line regimens are failing, by type of genotype

|  | Overall Prevalence* | GT1 | GT2 | GT3 | GT4 | GT5*** | GT6 |
| --- | --- | --- | --- | --- | --- | --- | --- |
| Nucleotide analogue (NS5B) | - | - | - | - | - |  | - |
| A150V | - | - | - | - | - |  | - |
| L159F | - | - | - | - | - |  | - |
| K206E | 1/122 | 0/15 | 0/1 | 1/100 | 0/4 |  | 0/2 |
| S282C/G/R/T | 1/122 | 0/15 | 0/1 | 1/100 | 0/4 |  | 0/2 |
| C316H/R | 1/122 | 1/15 | 0/1 | 0/100 | 0/4 |  | 0/2 |
| L320I/F/V | - | - | - | - | - |  | - |
| V321I/A | - | - | - | - | - |  | - |
| NS5A inhibitors (NS5A) | - | - | - | - | - |  | - |
| K24A/E/F/H/Q/R/S/T | 1/122 | 1/15 | 0/1 | 0/100 | 0/4 |  | 0/2 |
| K26E | - | - | - | - | - |  | - |
| M/F/L28A/C/I/G/K/L/M/S/T/V | 6/122 | 1/15 | 1/1 | 0/100 | 3/4 |  | 1/2 |
| P29R/S, del29 | - | - | - | - | - |  | - |
| Q/R/L/Q/A30C/D/E/G/H/K/L/N/P/Q/R/S/T/Y//G/H/R/S  Del30 | 22/122 | 5/15 | 0/1 | 13/100 | 4/4 |  | 0/2 |
| L31I/F/M/P/V/W | 14/122 | 7/15 | 0/1 | 3/100 | 3/4 |  | 1/2 |
| P32A/F/L/Q/ R/S del32 | - | - | - | - | - |  | - |
| S38F | - | - | - | - | - |  | - |
| H/P/T58A/C/D/G/H/L/N/P/S/R/T | 2/122 | 1/15 | 0/1 | 0/100 | 1/4 |  | 0/2 |
| Q/E62D/L | - | - | - | - | - |  | - |
| A/C/E92E/K/R/S/T/V/W | - | - | - | - | - |  | - |
| T/Y93A/C/F/H/L/N/R/S/T/W | 76/168 | 6/35 | 0/1 | 66/126 | 4/4 |  | 0/2 |

# **Table S15:** Mutation patterns among patients in whom first-line antiviral containing sofosbuvir and velpatasvir failed

**A total of 253 patients. *Data was only available for 168 patients and in some cases was only reported to a given region or mutation; -not detected; *** Not included in any of the available data

# **Table S16:** Mutation patterns among patients in whom first-line antiviral containing sofosbuvir and daclatasvir failed

|  | Overall Prevalence | GT1 | GT2 | GT3 | GT4 | GT5*** | GT6*** |
| --- | --- | --- | --- | --- | --- | --- | --- |
| Nucleotide analogue (NS5B) |  |  |  |  |  |  |  |
| A150V | - | - | - | - | - |  |  |
| L159F | 6/454 | 4/53 | 0/2 | 2/388 | 0/11 |  |  |
| K206E |  |  |  |  |  |  |  |
| S282C/G/R/T | 10/454 | 2/53 | 0/2 | 7/388 | 1/11 |  |  |
| C316H/R | 4/454 | 4/53 | 0/2 | 0/388 | 0/11 |  |  |
| L320I/F/V | - | - | - | - | - |  |  |
| V321I/A | - | - | - | - | - |  |  |
| NS5A inhibitors (NS5A) |  |  |  |  |  |  |  |
| K24A/E/F/H/Q/R/S/T | 1/465 | 0/53 | 1/2 | 0/400 | 0/10 |  |  |
| K26E | - | - | - | - | - |  |  |
| M/F/L28A/C/I/G/K/L/M/S/T/V | 6/465 | 1/53 | 0/2 | 0/400 | 5/10 |  |  |
| P29R/S, del29 | - | - | - | - | - |  |  |
| Q/R/L/Q/A30C/D/E/G/H/K/L/N/P/Q/R/S/T/Y//G/H/R/S  Del30 | 91/465 | 20/53 | 0/2 | 62/400 | 9/10 |  |  |
| L31I/F/M/P/V/W | 44/465 | 20/53 | 2/2 | 12/400 | 0/10 |  |  |
| P32A/F/L/Q/ R/S del32 | - | - | - | - | - |  |  |
| S38F | - | - | - | - | - |  |  |
| H/P/T58A/C/D/G/H/L/N/P/S/R/T | 3/465 | 1/53 | 0/2 | 0/400 | 2/10 |  |  |
| Q/E62D/L | - | - | - | - | - |  |  |
| A/C/E92E/K/R/S/T/V/W | - | - | - | - | - |  |  |
| T/Y93A/C/F/H/L/N/R/S/T/W | 245/465 | 29/53 | 0/2 | 211/400 | 5/10 |  |  |

**A total of 750 patients. *Data was only available for 465 patients and in some cases was only reported to a given region or mutation; -not detected; *** Not included in any of the available data

# **Table S17:** Mutation patterns among patients in whom first-line antiviral containing glecaprevir and pibrentasvir failed

|  | Overall Prevalence | GT1 | GT2 | GT3 | GT4*** | GT5*** | GT6*** |
| --- | --- | --- | --- | --- | --- | --- | --- |
| NS5A inhibitors (NS5A) |  |  |  |  |  |  |  |
| K24A/E/F/H/Q/R/S/T | 1/79 | 0/34 | 0/21 | 1/79 |  |  |  |
| K26E | - | - | - | - |  |  |  |
| M/F28A/C/I/G/K/L/M/S/T/V | 6/134 | 2/34 | 3/21 | 1/79 |  |  |  |
| P29R/S, del29 | - | - | - | - |  |  |  |
| L/Q/R/A30C/D/E/F/G/H/K/L/N/P/Q/R/S/T/Y  Del30 | 75/134 | 14/34 | 0/21 | 61/79 |  |  |  |
| L31I/F/M/P/V/W | 32/134 | 7/34 | 3/21 | 12/79 |  |  |  |
| P32A/F/L/Q/ R/S del32 | 2/134 | 2/34 | 0/21 | 0/79 |  |  |  |
| S38F | - | - | - | - |  |  |  |
| H/P/T58A/C/D/G/H/L/N/P/S/R/T | 13/134 | 12/34 | 0/21 | 1/79 |  |  |  |
| Q/E62D/L | - | - | - | - |  |  |  |
| A/E92C/E/K/R/S/T/V/W | - | - | - | - |  |  |  |
| T/Y93A/C/F/H/L/N/R/S/T/W | 85/147 | 20/45 | 0/21 | 65/81 |  |  |  |
| Protease inhibitors (NS3) | - | - | - | - |  |  |  |
| V36A/C/F/G/I/L/M | - | - | - | - |  |  |  |
| Q41K/R | - | - | - | - |  |  |  |
| F43I/L/S/V | - | - | - | - |  |  |  |
| T54A/C/G/S | - | - | - | - |  |  |  |
| V55A/I | - | - | - | - |  |  |  |
| Y56H/L/F | 13/134 | 3/34 | 0/21 | 10/79 |  |  |  |
| Q/L80H/K/L/R/Q | 7/134 | 5/34 | 0/21 | 2/79 |  |  |  |
| S122A/D/G/I/N/R/T | - | - | - | - |  |  |  |
| R155C/G/I/K/L/Q/M/S/T/W | - | - | - | - |  |  |  |
| A156G/H/K/L/P/S/T/V | 1/134 | 0/34 | 0/21 | 1/60 |  |  |  |
| V158I | - | - | - | - |  |  |  |
| A166S/T/Y | 3/134 | 0/34 | 0/21 | 3/60 |  |  |  |
| D168A/C/E/F/G/H/I/K/L/N/Q/R/T/V/Y | 9/134 | 0/34 | 0/21 | 9/79 |  |  |  |
| I/V170A/L/T | - | - | - | - |  |  |  |
| M175L | - | - | - | - |  |  |  |

**A total of 243 patients. *Data was only available for 147 patients and in some cases was only reported to a given region or mutation; -not detected; *** Not included in any of the available data

#

# **Table S18:** Mutation patterns among patients in whom second-line antiviral containing glecaprevir and pibrentasvir failed

|  | Overall Prevalence | GT1 | GT2** | GT3** | GT4** | GT5** | GT6** |
| --- | --- | --- | --- | --- | --- | --- | --- |
| NS5A inhibitors (NS5A) |  |  |  |  |  |  |  |
| K24A/E/F/H/Q/R/S/T | 4/34 | 4/34 |  |  |  |  |  |
| K26E | - | - |  |  |  |  |  |
| M/F28A/C/I/G/K/L/M/S/T/V | 12/34 | 12/34 |  |  |  |  |  |
| P29R/S, del29 | 1/34 | 1/34 |  |  |  |  |  |
| L/Q/R/A30C/D/E/F/G/H/K/L/N/P/Q/R/S/T/Y  Del30 | 18/34 | 18/34 |  |  |  |  |  |
| L31I/F/M/P/V/W | 11/34 | 11/34 |  |  |  |  |  |
| P32A/F/L/Q/ R/S del32 | 7/34 | 7/34 |  |  |  |  |  |
| S38F | - | - |  |  |  |  |  |
| H/P/T58A/C/D/G/H/L/N/P/S/R/T | 9/34 | 9/34 |  |  |  |  |  |
| Q/E62D/L | - | - |  |  |  |  |  |
| A/E92C/E/K/R/S/T/V/W | 2/34 | 2/34 |  |  |  |  |  |
| T/Y93A/C/F/H/L/N/R/S/T/W | 10/34 | 10/34 |  |  |  |  |  |
| Protease inhibitors (NS3) | - | - |  |  |  |  |  |
| V36A/C/F/G/I/L/M | 2/34 | 2/34 |  |  |  |  |  |
| Q41K/R | - | - |  |  |  |  |  |
| F43I/L/S/V | - | - |  |  |  |  |  |
| T54A/C/G/S | - | - |  |  |  |  |  |
| V55A/I | - | - |  |  |  |  |  |
| Y56H/L/F | 1/34 | 1/34 |  |  |  |  |  |
| Q/L80H/K/L/R/Q | - | - |  |  |  |  |  |
| S122A/D/G/I/N/R/T | 1/34 | 1/34 |  |  |  |  |  |
| R155C/G/I/K/L/Q/M/S/T/W | 5/34 | 5/34 |  |  |  |  |  |
| A156G/H/K/L/P/S/T/V | 12/34 | 12/34 |  |  |  |  |  |
| V158I | - | - |  |  |  |  |  |
| A166S/T/Y | - | - |  |  |  |  |  |
| D168A/C/E/F/G/H/I/K/L/N/Q/R/T/V/Y | 9/34 | 9/34 |  |  |  |  |  |
| I/V170A/L/T | - | - |  |  |  |  |  |
| M175L | - | - |  |  |  |  |  |

; *** Not included in any of the available data

# **Table S19:** Mutation patterns among patients in whom second-line antiviral containing sofosbuvir/velpasavir /voxilaprevir failed

|  | Overall Prevalence | GT1 | GT2 | GT3 | GT4 | GT5*** | GT6*** |
| --- | --- | --- | --- | --- | --- | --- | --- |
| Nucleotide analogue (NS5B) |  |  |  |  |  |  |  |
| A150V | 1/62 | 0/38 | - | 1/22 | 0/2 |  |  |
| L159F | 1/62 | 1/38 | - | 0/22 | 0/2 |  |  |
| K206E |  | - | - | - | - |  |  |
| S282C/G/R/T |  | - | - | - | - |  |  |
| C316H/R | 1/62 | 1/38 |  | 0/26 | 0/2 |  |  |
| L320I/F/V |  | - | - | - | - |  |  |
| V321I/A | 1/62 | 0/38 |  | 1/26 | 0/2 |  |  |
| NS5A inhibitors (NS5A) |  |  |  |  |  |  |  |
| K24A/E/F/H/Q/R/S/T | 1/71 | 1/43 |  | 0/26 | 0/2 |  |  |
| K26E |  | - | - | - | - |  |  |
| M/F/L28A/C/I/G/K/L/M/S/T/V | 5/71 | 5/43 |  | 0/26 | 0/2 |  |  |
| P29R/S, del29 |  | - | - | - | - |  |  |
| Q/R/L/Q/A30C/D/E/G/H/K/L/N/P/Q/R/S/T/Y//G/H/R/S  Del30 | 19/71 | 13/43 |  | 6/26 | 0/2 |  |  |
| L31I/F/M/P/V/W | 20/71 | 17/43 |  | 1/26 | 2/2 |  |  |
| P32A/F/L/Q/ R/S del32 |  | - | - | - | - |  |  |
| S38F |  | - | - | - | - |  |  |
| H/P/T58A/C/D/G/H/L/N/P/S/R/T |  | - | - | - | - |  |  |
| Q/E62D/L |  | - | - | - | - |  |  |
| A92E/K/T/V |  | - | - | - | - |  |  |
| E/C92K/R/S/T/W |  | - | - | - | - |  |  |
| T/Y93A/C/F/H/L/N/R/S/T/W | 38/71 | 14/43 |  | 22/26 | 2/2 |  |  |
| Protease inhibitors (NS3) |  | - | - | - | - |  |  |
| V36A/C/F/G/I/L/M | 2/71 | 2/43 |  | 0/26 | 0/2 |  |  |
| Q41K/R | 1/71 | 1/43 |  | 0/26 | 0/2 |  |  |
| F43I/L/S/V |  | - | - | - | - |  |  |
| T54A/C/G/S | 2/71 | 2/43 |  | 0/26 | 0/2 |  |  |
| V55A/I | 1/71 | 1/43 |  | 0/26 | 0/2 |  |  |
| Y56H/L/F | 5/71 | 4/43 |  | 1/26 | 0/2 |  |  |
| L/Q80H/K/L/R/Q | 13/71 | 13/43 |  | 0/26 | 0/2 |  |  |
| S122A/D/G/I/N/R/T |  | - | - | - | - |  |  |
| R155C/G/I/K/L/Q/M/S/T/W |  | - | - | - | - |  |  |
| A156G/H/K/L/P/S/T/V | 1/71 | 0/43 |  | 0/26 | 1/2 |  |  |
| V158I |  | - | - | - | - |  |  |
| A166S/T/Y | 2/71 | 0/43 |  | 2/26 | 0/2 |  |  |
| D168A/C/E/F/G/H/I/K/L/N/Q/R/T/V/Y | 10/71 | 4/43 |  | 5/26 | 1/2 |  |  |
| I/V170A/L/T |  | - | - | - | - |  |  |
| M175L |  | - | - | - | - |  |  |

**A total of 94 patients. *Data was only available for 71 patients and in some cases was only reported to a given region or mutation; -not detected; *** Not included in any of the available data

**References**

1 Munn Z, Moola S, Lisy K, Riitano D, Tufanaru C. Methodological guidance for systematic reviews of observational epidemiological studies reporting prevalence and cumulative incidence data. *Int J Evid Based Healthc* 2015; 13: 147–53.

2 Quinn N, Bergin C, Bannan C, *et al.* Viral hepatitis C: Therapy and resistance Retreatment of patients experiencing failure with Hepatitis C direct-acting antivirals. Sof/Vel/Vox, G/P+Sof. In: European Association for the Study of the Liver. Vienna, Austria, 2023. https://www.natap.org/2023/EASL/EASL_05.htm (accessed September 14, 2023).

3 Xie Z, Deng K, Xia Y, *et al.* Efficacy and safety of direct-acting antiviral therapies and baseline predictors for treatment outcomes in hepatitis C patients: A multicenter, real-world study in Guangdong, China. *J Med Virol* 2022; 94: 4459–69.

4 Mushtaq S, Hashmi AH, Khan A, Asad Raza Kazmi SM, Manzoor S. Emergence and Persistence of Resistance-Associated Substitutions in HCV GT3 Patients Failing Direct-Acting Antivirals. *Front Pharmacol* 2022; 13: 894460.

5 Onofrio FQ, Cooper C, Borgia SM, *et al.* Salvage Therapy with Sofosbuvir/Velpatasvir/Voxilaprevir in DAA-experienced Patients: Results from a Prospective Canadian Registry. *Clin Infect Dis an Off Publ Infect Dis Soc Am* 2021; 72: e799–805.

6 Wang GP, Schnell GL, Kort JJ, *et al.* Linkage of resistance-associated substitutions in GT1 sofosbuvir + NS5A inhibitor failures treated with glecaprevir/pibrentasvir. *J Hepatol* 2021; 75: 820–8.

7 Jia Y, Yue W, Gao Q, *et al.* Characterization of a Novel Hepatitis C Subtype, 6xj, and Its Consequences for Direct-Acting Antiviral Treatment in Yunnan, China. *Microbiol Spectr* 2021; 9: e0029721.

8 Di Maio VC, Barbaliscia S, Teti E, *et al.* Resistance analysis and treatment outcomes in hepatitis C virus genotype 3-infected patients within the Italian network VIRONET-C. *Liver Int Off J Int Assoc Study Liver* 2021; 41: 1802–14.

9 Isfordink CJ, van de Laar TJW, Rebers SPH, *et al.* Direct-Acting Antiviral Treatment for Hepatitis C Genotypes Uncommon in High-Income Countries: A Dutch Nationwide Cohort Study. *Open forum Infect Dis* 2021; 8: ofab006.

10 Di Stefano M, Faleo G, Farhan Mohamed AM, *et al.* Resistance Associated Mutations in HCV Patients Failing DAA Treatment. *New Microbiol* 2021; 44: 12–8.

11 Fourati S, Rodriguez C, Soulier A, *et al.* Fitness-associated substitutions following failure of direct-acting antivirals assessed by deep sequencing of full-length hepatitis C virus genomes. *Aliment Pharmacol Ther* 2020; 52: 1583–91.

12 Dietz J, Kalinina O V, Vermehren J, *et al.* Resistance-associated substitutions in patients with chronic hepatitis C virus genotype 4 infection. *J Viral Hepat* 2020; 27: 974–86.

13 Douglas MW, Tay ESE, Wang D Sen, *et al.* Impact of an Open Access Nationwide Treatment Model on Hepatitis C Virus Antiviral Drug Resistance. *Hepatol Commun* 2020; 4: 904–15.

14 Alessio L, Onorato L, Sangiovanni V, *et al.* DAA-based treatment for HIV-HCV-coinfected patients: analysis of factors of sustained virological response in a real-life study. *Antivir Ther* 2020; 25: 193–201.

15 Fourati S, Rodriguez C, Hézode C, *et al.* Frequent Antiviral Treatment Failures in Patients Infected With Hepatitis C Virus Genotype 4, Subtype 4r. *Hepatology* 2019; 69: 513–23.

16 Gozlan Y, Bucris E, Shirazi R, *et al.* High frequency of multiclass HCV resistance-associated mutations in patients failing direct-acting antivirals: real-life data. *Antivir Ther* 2019; 24: 221–8.

17 Di Maio VC, Aragri M, Masetti C, *et al.* Italian real life experience of resistance guided retreatment in HCV infected patients who previously failed a NS5A inhibitor containing regimen. In: AASLD. Boston, MA, USA, 2019. https://www.natap.org/2019/AASLD/AASLD_87.htm (accessed September 12, 2023).

18 Dietz J, Vermehren J, Peiffer K-H, *et al.* Comparison of HCV resistance-associated substitutions in patients infected with HCV genotype 3 before and after failure to DAA combination therapies. In: European Association for the Study of the Liver. Vienna, Austria, 2019. https://www.natap.org/2019/EASL/EASL_79.htm (accessed September 14, 2023).

19 Rodrigues JPV, Campos GRF, Bittar C, *et al.* Selection dynamics of HCV genotype 3 resistance-associated substitutions under direct-acting antiviral therapy pressure. *Brazilian J Infect Dis an Off Publ Brazilian Soc Infect Dis* 2022; 26: 102717.

20 Elhence A, Singh A, Anand A, *et al.* Real-world re-treatment outcomes of direct-acting antiviral therapy failure in patients with chronic hepatitis C. *J Med Virol* 2021; 93: 4982–91.

21 Fernandes Campos GR, Ward J, Chen S, *et al.* A novel substitution in NS5A enhances the resistance of hepatitis C virus genotype 3 to daclatasvir. *J Gen Virol* 2021; 102. DOI:10.1099/jgv.0.001496.

22 Ruiz I, Fourati S, Ahmed-Belkacem A, *et al.* Real-world efficacy and safety of direct-acting antiviral drugs in patients with chronic hepatitis C and inherited blood disorders. *Eur J Gastroenterol Hepatol* 2021; 33: e191–6.

23 Fedorchenko S V, Martynovych T, Klimenko Z, Yanchenko V, Solianyk I. Retreatment of patients with chronic hepatitis C, subtype 1b and cirrhosis, who failed previous direct-acting antiviral therapy including first- and second-generation NS5A inhibitors with ombitasvir/paritaprevir/ritonavir, dasabuvir + sofosbuvir + ribavi. *J Viral Hepat* 2020; 27: 548–51.

24 Chen Q, Perales C, Soria ME, *et al.* Deep-sequencing reveals broad subtype-specific HCV resistance mutations associated with treatment failure. *Antiviral Res* 2020; 174: 104694.

25 Minosse C, Selleri M, Giombini E, *et al.* Clinical and virological properties of hepatitis C virus genotype 4 infection in patients treated with different direct-acting antiviral agents. *Infect Drug Resist* 2018; 11: 2117–27.

26 Dietz J, Susser S, Vermehren J, *et al.* Patterns of Resistance-Associated Substitutions in Patients With Chronic HCV Infection Following Treatment With Direct-Acting Antivirals. *Gastroenterology* 2018; 154: 976-988.e4.

27 Bachofner J, Valli P V, Bergamin I, *et al.* Excellent outcome of direct antiviral treatment for chronic hepatitis C in Switzerland. *Swiss Med Wkly* 2018; 148: w14560.

28 Salmon D, Trimoulet P, Gilbert C, *et al.* Factors associated with DAA virological treatment failure and resistance-associated substitutions description in HIV/HCV coinfected patients. *World J Hepatol* 2018; 10: 856–66.

29 Paolucci S, Premoli M, Novati S, *et al.* Baseline and Breakthrough Resistance Mutations in HCV Patients Failing DAAs. *Sci Rep* 2017; 7: 16017.

30 Dietz J, Müllhaupt B, Buggisch P, *et al.* Long-term persistence of HCV resistance-associated substitutions after DAA treatment failure. *J Hepatol* 2023; 78: 57–66.

31 D’Ambrosio R, Pasulo L, Puoti M, *et al.* Real-world effectiveness and safety of glecaprevir/pibrentasvir in 723 patients with chronic hepatitis C. *J Hepatol* 2019; 70: 379–87.

32 de Salazar A, Dietz J, di Maio VC, *et al.* Prevalence of resistance-associated substitutions and retreatment of patients failing a glecaprevir/pibrentasvir regimen. *J Antimicrob Chemother* 2020; 75: 3349–58.

33 Pearlman B, Perrys M, Hinds A. Sofosbuvir/Velpatasvir/Voxilaprevir for Previous Treatment Failures With Glecaprevir/Pibrentasvir in Chronic Hepatitis C Infection. *Am J Gastroenterol* 2019; 114: 1550–2.

34 Flamm S, Mutimer D, Asatryan A, *et al.* Glecaprevir/Pibrentasvir in patients with chronic HCV genotype 3 infection: An integrated phase 2/3 analysis. *J Viral Hepat* 2019; 26: 337–49.

35 Poordad F, Pol S, Asatryan A, *et al.* Glecaprevir/Pibrentasvir in patients with hepatitis C virus genotype 1 or 4 and past direct-acting antiviral treatment failure. *Hepatology* 2018; 67: 1253–60.

36 Lok AS, Sulkowski MS, Kort JJ, *et al.* Efficacy of Glecaprevir and Pibrentasvir in Patients With Genotype 1 Hepatitis C Virus Infection With Treatment Failure After NS5A Inhibitor Plus Sofosbuvir Therapy. *Gastroenterology* 2019; 157: 1506-1517.e1.

37 Mawatari S, Oda K, Kumagai K, *et al.* Viral and host factors are associated with retreatment failure in hepatitis C patients receiving all-oral direct antiviral therapy. *Hepatol Res* 2020; 50: 453–65.

38 Uemura H, Uchida Y, Kouyama J-I, *et al.* NS5A-P32 deletion as a factor involved in virologic failure in patients receiving glecaprevir and pibrentasvir. *J Gastroenterol* 2019; 54: 459–70.

39 Sezaki H, Suzuki F, Hosaka T, *et al.* Initial- and re-treatment effectiveness of glecaprevir and pibrentasvir for Japanese patients with chronic hepatitis C virus-genotype 1/2/3 infections. *J Gastroenterol* 2019; 54: 916–27.

40 Osawa M, Imamura M, Teraoka Y, *et al.* Real-world efficacy of glecaprevir plus pibrentasvir for chronic hepatitis C patient with previous direct-acting antiviral therapy failures. *J Gastroenterol* 2019; 54: 291–6.

41 Smith DA, Bradshaw D, Mbisa JL, *et al.* Real world SOF/VEL/VOX retreatment outcomes and viral resistance analysis for HCV patients with prior failure to DAA therapy. *J Viral Hepat* 2021; 28: 1256–64.

42 Garcia-Cehic D, Rando A, Rodriguez-Frias F, *et al.* Resistance-associated substitutions after sofosbuvir/velpatasvir/voxilaprevir triple therapy failure. *J Viral Hepat* 2021; 28: 1319–24.

43 Dietz J, Di Maio VC, de Salazar A, *et al.* Failure on voxilaprevir, velpatasvir, sofosbuvir and efficacy of rescue therapy. *J Hepatol* 2021; 74: 801–10.

44 Degasperi E, Spinetti A, Lombardi A, *et al.* Real-life effectiveness and safety of sofosbuvir/velpatasvir/voxilaprevir in hepatitis C patients with previous DAA failure. *J Hepatol* 2019; 71: 1106–15.

45 Wilson E, Covert E, Hoffmann J, *et al.* A pilot study of safety and efficacy of HCV retreatment with sofosbuvir/velpatasvir/voxilaprevir in patients with or without HIV (RESOLVE STUDY). *J Hepatol* 2019; 71: 498–504.

46 Sarrazin C, Cooper CL, Manns MP, *et al.* No impact of resistance-associated substitutions on the efficacy of sofosbuvir, velpatasvir, and voxilaprevir for 12 weeks in HCV DAA-experienced patients. *J Hepatol* 2018; 69: 1221–30.

47 Bourlière M, Gordon SC, Schiff ER, *et al.* Deferred treatment with sofosbuvir-velpatasvir-voxilaprevir for patients with chronic hepatitis C virus who were previously treated with an NS5A inhibitor: an open-label substudy of POLARIS-1. *lancet Gastroenterol Hepatol* 2018; 3: 559–65.

48 Lawitz E, Poordad F, Wells J, *et al.* Sofosbuvir-velpatasvir-voxilaprevir with or without ribavirin in direct-acting antiviral-experienced patients with genotype 1 hepatitis C virus. *Hepatology* 2017; 65: 1803–9.

49 Gozlan Y, Bucris E, Shirazi R, *et al.* The impact of treatment emerging resistance associated substitutions on outcomes of re-treatment using new generation HCV treatments: Nation-wide real-world analysis. In: European Association for the Study of the Liver. Vienna, Austria, 2020. https://www.postersessiononline.eu/173580348_eu/congresos/VHC2020/aula/-P03_5_VHC2020.pdf (accessed September 14, 2024).
